# Ligand-Specific Nano-Contrast Agents Promote Enhanced Breast Cancer CT Detection at 0.5 mg Au

**DOI:** 10.3390/ijms23179926

**Published:** 2022-09-01

**Authors:** Kalyan Ramesh, Alice Truong, Yuzhen Wang, Mary Rusckowski, Manos Gkikas

**Affiliations:** 1 Department of Chemistry, University of Massachusetts Lowell, Lowell, MA 01854, USA; 2 Department of Radiology, University of Massachusetts Chan Medical School, Worcester, MA 01655, USA

**Keywords:** X-ray contrast agents, breast cancer, computed tomography, nanotechnology, ligand-specific probes, spectral CT, dual-energy CT, molecular imaging, medical diagnostics, molecular recognition

## Abstract

For many cancer types, being undetectable from early symptoms or blood tests, or often detected at late stages, medical imaging emerges as the most efficient tool for cancer screening. MRI, ultrasound, X-rays (mammography), and X-ray CT (CT) are currently used in hospitals with variable costs. Diagnostic materials that can detect breast tumors through molecular recognition and amplify the signal at the targeting site in combination with state-of-the-art CT techniques, such as dual-energy CT, could lead to a more precise detection and assist significantly in image-guided intervention. Herein, we have developed a ligand-specific X-ray contrast agent that recognizes α5β1 integrins overexpressed in MDA-MB-231 breast cancer cells for detection of triple (−) cancer, which proliferates very aggressively. In vitro studies show binding and internalization of our nanoprobes within those cells, towards uncoated nanoparticles (NPs) and saline. In vivo studies show high retention of ~3 nm ligand-PEG-S-AuNPs in breast tumors in mice (up to 21 days) and pronounced CT detection, with statistical significance from saline and iohexol, though only 0.5 mg of metal were utilized. In addition, accumulation of ligand-specific NPs is shown in tumors with minimal presence in other organs, relative to controls. The prolonged, low-metal, NP-enhanced spectral-CT detection of triple (−) breast cancer could lead to breakthrough advances in X-ray cancer diagnostics, nanotechnology, and medicine.

## 1. Introduction

Breast cancer along with colorectal and lung cancer contribute to ~44% of all gynecologic cancers and significantly affect women’s health [1,2,3,4,5,6,7]. Achieving early cancer detection is one of the greatest challenges in medicine since it increases the chances for successful treatment and cure [4]. Many cancer types, however, are undetectable from early symptoms or blood tests (including micro-tumors), and not detected until later stages, where medical imaging (MRI, ultrasound, mammography: low-energy X-rays, and X-ray CT: computed tomography) emerges as the most efficient tool for screening [8,9,10,11,12,13,14,15]. From these, ultrasound, mammography, and CT are considered the most suitable in terms of patient comfort and cost (CT is ~half the cost of an MRI). Diagnostic X-ray contrast agents that can detect breast cancer early through molecular recognition of breast cancer cells, in combination with spectral CT, a form of CT that allows for visualization of tissue composition based on intensity of signal, which is dictated by atomic number and density of materials, and thus is enhanced by high Z-metals, could amplify the signal at the site of tumor, thus leading to a more precise breast cancer detection, and assist significantly in image-guided intervention.

Metal-based molecular probes are used to provide X-ray contrast clinically [16]. Small tri-iodinated molecules such as sodium diatrizoate, iohexol (Omnipaque), and others have been approved by the FDA for abdominal imaging, as well as for angiography. These contrast agents work very efficiently in detecting abnormalities, e.g., blocked arteries, are compatible with most cells, and excrete through kidneys within 10–15 min. However, they lack target specificity, may cause renal toxicity, especially in patients with kidney-related issues, and cannot provide long-life imaging, as needed for non-invasive disease monitoring, in addition to the large amount required for CT imaging (~30 g of I: iodine for oral administration in humans). Efforts to encapsulate iodine-based contrast agents in ~100 nm carriers, such as polymeric micelles [17] or liposomes [18,19,20,21,22,23,24], have extended their biological clearance for venous imaging/X-ray signal enhancement in preclinical models, leading to alternative clearance paths compared to conventional, contrast media, with achieved metal loadings of 22–155 mg I/mL [17,18,19,20,21,22,23,24], and contrast amount injected in mice of 40–60 mg I [17,18,19,20,21,22,23,24].

On the other hand, high atomic number (high Z) metal-based nanomaterials including bismuth [25], tantalum [26,27,28], lanthanides [26,27], and gold [23,24,29,30,31,32,33,34,35,36,37,38,39,40,41] have been utilized preclinically. These materials have significant advantages over iodine-based agents, including higher density (19.6 g/mL for gold: Au, compared to 4.9 g/mL of I and 1.6 g/mL of Ca: calcium), higher attenuation coefficient and K-edge (81 keV for Au, compared to 33 keV of I and 4 keV of Ca), and offer prolonged circulation for vascular CT imaging [23,24,29,30,31,32,33,34], due to alternative clearance paths (related mostly to NP size). Metal loadings of 3 mg Au/mL (intraperitoneal injection) [37], 75 mg Au/mL (intravenous: iv) [24], 200 mg Au/mL (iv) [23], and 134–270 mg Au/mL (iv) [29,30] have been reported, with contrast amount injected in mice (~20 g weight) of 5–54 mg Au [23,24,29,31,32,36,38,40], and 38–67 mg Au in rats [30]. However, many of the contrast agents above did not provide high CT contrast (defined as clearly discernible signal from tissue/tumor) at the reported amounts, possibly due to distribution to various organs. The majority of preclinical contrast-enhanced CT studies [23,24,29,30,31,32,33,34] have focused on polymer-coated nanoparticle (NP) fate and clearance post iv injection in naïve animals, or on passive tumor imaging (using vascularly injected blood pool agents) in cancer models. A few cases have reported on ligand-specific, disease-targeting NPs [35,36,37,38,39,40,41].

Since X-ray absorption is highly dependent on K-edge and density of materials, metallic nano-contrast agents carrying specific cell receptor-targeting moieties emerge as very promising CT contrast probes for detecting and monitoring diseases such as cancer, since the targeting moieties on NPs can reduce off-target effects and assist in tumor diagnosis. Empowering the detection with advanced, state-of-the-art CT techniques, such as dual-energy CT (DE-CT) [23,24] or photon-counting spectral CT (PCD-CT) [26,27], could allow for diagnosis with higher precision, due to metal-enhancement of the signal, lower noise, and higher contrast resolution, permitting for the development of the next generation of CT-guided cancer diagnostics. In this paper, we focus on prolonged detection of triple (−) breast cancer at sub-mg NP dosages, using poly(ethylene glycol) (PEG)-stabilized AuNPs conjugated with a fibronectin–mimetic peptide [42,43,44,45], reported to recognize and bind specifically to α5β1 integrins overexpressed in MDA-MB-231 breast cancer cells. This type of breast cancer accounts for 15% of breast tumors that do not express hormone receptors such as estrogen, progesterone, or human epidermal growth factor receptor 2 (HER2); it proliferates very aggressively, and is hard to be completely eliminated [46]. We report on the synthesis of a targeted X-ray contrast agent, its adhesion/internalization to MDA-MB-231 breast cancer cells, and finally explore the signal retention in tumor in a cancer mouse model for up to 21 days. Precise location of ligand-specific NPs in tumors/micro-tumors could lead to their elimination with the assistance of radiation therapy, chemo/immuno-therapy, or NIR thermal ablation. Utilization of a small amount of metal (contrast agent) reassures minimal negative impact on normal cells, low in vivo toxicity, and nanomaterial retention on target.

## 2. Results and Discussion

### 2.1. Dodecanethiol-Coated Gold Nanoparticles (C12-S-AuNPs)

Hydrophobic C12-S-AuNPs were used in this study as a precursor for ligand exchange with a thiol-terminated hydrophilic polymer. UV–Vis results of C12-S-AuNPs in dichloromethane (DCM) at 0.04 mg/mL showed a surface plasmon resonance (SPR) peak at 516 nm (Figure S1). The morphology and size of the synthesized C12-S-AuNPs were studied by transmission electron microscopy (TEM), showing a spherical shape and an average size of 3.7 ± 0.6 nm (Figure S2). Thermogravimetric analysis (TGA) results showed ~11 ± 0 wt% dodecanethiol incorporation (decomposition temperature: *T_d_ =* 250 ± 1 °C) onto the NP surface, leading to a metal content of 89 wt% Au (Figure S3).

### 2.2. Synthesis of Hydrophilic PEG-Coated Gold Nanoparticles (x-PEG-S-AuNPs)

**Scheme 1:**
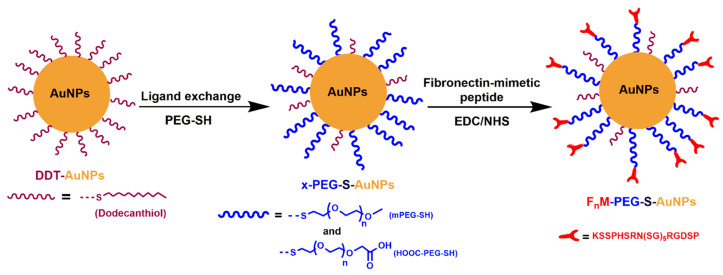
Synthesis of mPEG-S-AuNPs and F_n_M-S-PEG-AuNPs contrast agents.

Two hydrophilic probes, symbolized as x-PEG-S-AuNPs, were then synthesized by ligand exchange of C12-S-AuNPs with excess mPEG-SH or HOOC-PEG-SH (Scheme 1), followed by precipitation in hexane to remove liberated dodecanethiol. In addition, high speed centrifugation (fractionation) was utilized to remove unbound PEG-SH, since free polymeric chains (supernatant) have been found to co-exist with polymer-coated NPs (pellet) without this purification step [47]. The PEG coating favors solubility/dispersibility due to hydration of the NP core, reduces non-specific binding and protein adsorption, and enhances the biocompatibility and half-life of the NP probe, providing stealth properties in the bloodstream [30,48]. To reassure that PEG-SH has not been oxidized by air (which could result in less available polymer free-thiol for NP conjugation), size exclusion chromatography (SEC) was performed. Results showed monomodal distributions for the PEG polymers with calculated molecular weights (*M*_n_) of 6400 g/mol and 5500 g/mol for mPEG-SH and HOOC-PEG-SH, respectively, and dispersities (*Ð*) of 1.20 and 1.19 (Figure S4). The collected x-PEG-S-AuNPs were highly soluble/dispersible in water (depending on the concentration utilized), unlike the C12-S-AuNPs precursor.

**Figure 1:**
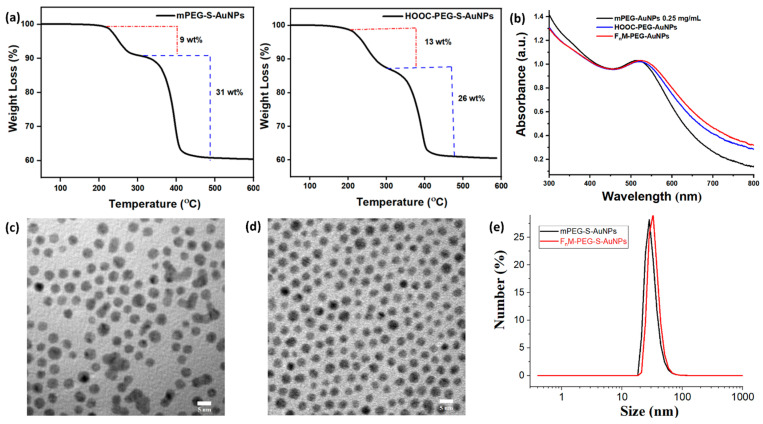
(**a**) TGA curves of mPEG-S-AuNPs and HOOC-S-AuNPs; (**b**) UV spectra of mPEG-S-AuNPs, HOOC-S-AuNPs, and F_n_M-PEG-S-AuNPs at 0.25 mg/mL in water; (**c**) TEM image of mPEG-S-AuNPs and (**d**) F_n_M-PEG-S-AuNPs; (**e**) DLS spectra of the two hydrophilic probes at 0.20 mg/mL in water.

TGA was performed to determine the grafting density of the polymer onto the surface of the hydrophilic NP probes. Results of mPEG-S-AuNPs showed two weight losses: one between 50 and 300 °C (*T_d_ =* 247 ± 2 °C), which we attribute to a fraction of C12-SH still attached onto the NP core (9 ± 0 wt% incorporation), and another one between 300 and 500 °C (*T_d_ =* 396 ± 1 °C) with 31 ± 1 wt% loss, which we attribute to bound PEG-SH (Figure 1a; analytical results in Figure S5; mPEG-SH has a *T_d_ =* 404 ± 2 °C, Figure S6), denoting a 60 wt% Au content. On the other hand, HOOC-PEG-S-AuNPs had a 1st weight loss of 13 ± 0 wt% (*T_d_ =* 258 ± 3 °C) and a 2nd weight loss of 26 ± 0 wt% (*T_d_ =* 403 ± 2 °C), leading also to a 60 wt% Au content (Figure 1a; analytical results in Figure S7; HOOC-PEG-SH has a *T_d_ =* 406 ± 1 °C, Figure S8). Overall, both x-PEG-S-AuNP contrast agents had a metal content of 60%. We would like to postulate that due to frequent examination of Au concentration with atomic absorption spectroscopy, the grafted wt% of stabilizer onto the NP core is often not reported. This is important since it can change the NP hydrophilicity, non-specific binding, and the interaction with proteins. In addition, preparation of non matrix-free nanocomposites (where the additional purification/fractionation step has not been performed [47]) could lead to a mixture of free polymer (e.g., PEG-SH) and PEG-S-AuNPs, and that could affect the material in vivo biodistribution, since a smaller amount of actually conjugated polymer onto the NPs could induce NP aggregation with other problems to arise.

UV−Vis absorption spectra were recorded to determine the SPR bands of the hydrophilic X-ray probes. mPEG-S-AuNPs and HOOC-PEG-S-AuNPs had absorption peaks at 520 nm and 524 nm in water at 0.25 mg/mL, respectively (Figure 1b), very close to the lipophilic C12-S-AuNPs (peak at 516 nm in DCM; different solvents lead to different refractive indices; Figure S1), denoting a similar core size after ligand exchange. TEM results for the two hydrophilic probes showed average Au core sizes of 3.4 ± 0.5 nm for mPEG-S-AuNPs (Figure 1c; analytical results in Figure S9) and 3.1 ± 0.5 nm for HOOC-PEG-S-AuNPs (Figure S10), confirming no change in core size post polymer conjugation (3.7 ± 0.6 nm for C12-S-AuNPs; Figure S3). Dynamic light scattering (DLS) results for mPEG-S-AuNPs in water at 0.2 mg/mL showed a number-average hydrodynamic size of 30.1 ± 3.6 nm (Figure 1e; the size was the same even when examined at 0.1 or 0.5 mg/mL), which correlates well with larger dynamic size in solution due to the conjugated PEG polymer. A similar increase in size due to polymer attachment has been reported by Cai et al. [31] and the group of Badea [24].

### 2.3. Synthesis of Ligand-Specific PEG-Gold Nanoparticles (F_n_M-PEG-S-AuNPs)

**Figure 2:**
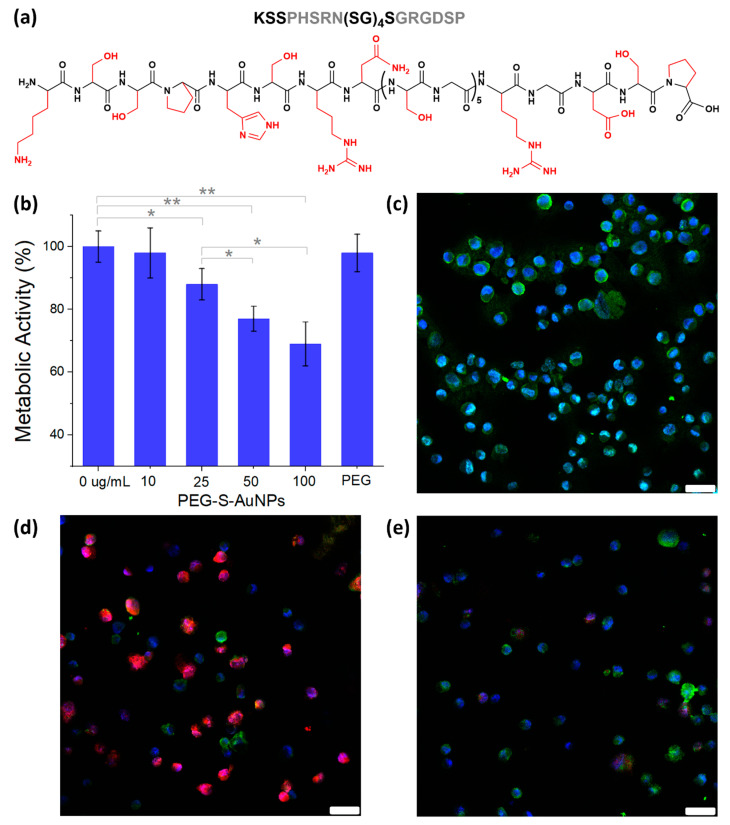
(**a**) The F_n_M-peptide utilized. (**b**) Cell metabolic activity of mPEG-S-AuNPs with 3T3 mouse fibroblasts. (**c**–**e**) Fluorescence studies of MDA-MB-231 human breast cancer cells incubated at 1:1 volume ratio with (**c**) L-15 medium only and two dyes, (**d**) medium supplemented with ~3 nm F_n_M-S-PEG-AuNPs at 250 μg Au/mL, and (**e**) medium only and three dyes utilized at the same conditions. Dyes used were Alexa Fluor™ 594 NHS (*pseudo-red*) for NP peptide staining, wheat germ agglutinin-Alexa Fluor™ 488 conjugate (*pseudo-green*) for cell membrane staining, and DAPI (*pseudo-blue*) for nuclei staining. Two washing cycles per dye were performed. Scale bar is 25 μm. Statistical significances are given as follows: * *p* < 0.05 and ** *p* < 0.01.

The fibronectin-mimetic (F_n_M) peptide KSSPHSRN(SG)_5_RGDSP (Figure 2a), which has been shown to bind specifically to α5β1 integrins overexpressed in MDA-MB-231 breast cancer cells in vitro and in vivo [42,45] was covalently conjugated to HOOC-PEG-S-AuNPs in the presence of EDC/NHS, yielding KSSPHSRN(SG)_5_RGDSP-PEG-S-AuNPs. The F_n_M-nanoprobe was engineered to specifically recognize triple (−) breast tumors, and potentially amplify the CT contrast (non-invasively) in relevant animal models. UV–Vis results in water at 0.25 mg/mL showed an SPR peak at 526 nm (Figure 1b), close to that of the HOOC-S-AuNPs precursor. TEM results of F_n_M-PEG-S-AuNPs showed an average Au core size of 3.1 ± 0.5 nm (Figure 1d; analytical results in Figure S11), while DLS results at 0.2 mg/mL revealed a hydrodynamic number-average size of 39.8 ± 8.8 nm (Figure 1e), slightly higher than the precursor due to the anchoring of the peptide. The NPs could be completely redispersed from solid/powder form within 5–10 min of sonication and were stable in aqueous solutions at 4 °C for more than 7 months (SPR peak remained unchanged); they even showed stability at 10% NaCl (Figure S12). To quantify the amount of peptide conjugated onto the NPs, a calibration curve of the three most prevalent amino acids of the F_n_M peptide, e.a. Ser (9), Gly (6), and Pro (2), was built between 0 and 50 μM after peptide or NP–peptide hydrolysis (Figures S13–S15) and the amino acids were detected by liquid chromatography–mass spectroscopy (LC-MS). Results showed 36, 26, and 30 μmol of F_n_M-peptide per 6 mg of NP for Ser, Gly, and Pro. Assuming average values for the three amino acids, the F_n_M-PEG-S-AuNPs had ~5 μmol of peptide/mg of NP, while mPEG-S-AuNPs had 0 μmol of peptide/mg of NP.

### 2.4. Fibroblasts Metabolic Activity and Binding Studies with MDA-MB-231 Breast Cancer Cells

First, we tested the in vitro compatibility of our mPEG-S-AuNPs with fibroblasts at different concentrations, by exploring the cell metabolic activity, which is considered an indirect estimation of cell viability. Fibroblasts were used as a normal cell line (dermal) to examine the cytotoxicity of our NPs. Cultured 3T3 fibroblasts in Dulbecco’s Modified Eagle Medium (DMEM) supplemented with 10% fetal bovine serum (FBS) at 37 °C were grown at 1–2 × 10^5^ cells/mL in 5% CO_2_, and then mixed at 1:1 volume ratio with PEG-S-AuNPs at 0–100 μg Au/mL, followed by 24 h incubation. No difference in metabolic activity (alamarBlue assay) was observed for the PEG stabilizer alone (~98%), while 98–69% of metabolic activity was observed between 10 and 100 μg Au/mL in PEG-stabilized AuNPs (Figure 2b). Au concentrations between 10 < x < 25 μg/mL seemed to fully retain the metabolic activity, showing safer cytocompatibility profiles. At higher Au concentrations, there was a trend of decreasing metabolic activity by concentration, though without exceeding the fibroblasts’ half maximal inhibitory concentration (IC50). Overall, PEG-stabilized AuNPs showed safe viability profiles with 3T3 cells up to 100 μg Au/mL.

We then confirmed binding of our ligand-specific contrast NPs in breast cancer cells by both optical and fluorescence microscopy. As shown in Figure S16, the F_n_M-PEG-S-AuNPs showed high adhesion to MDA-MB-231 cells at 250 μg Au/mL after 24 h incubation at 1:1 volume and three PBS washings, compared to MDA-MB-231 cells incubated with media only (Figure S17). On the other hand, mPEG-S-AuNPs showed lower adhesion at the same concentration (Figure S18). The ligand-specific NPs were also shown to internalize in MDA-MB-231 cells by fluorescence microscopy (Figure 2d). Conjugation of F_n_M-PEG-S-AuNPs with Alexa Fluor™ 594 NHS (ex./em. at 590/617 nm) revealed enhanced *pseudo-red* signal inside MDA-MB-231 cells after incubation (Figure 2d; 250 μg Au/mL were used) over control (cells only), where mainly *pseudo-green* (wheat germ agglutinin-Alexa Fluor™ 488 conjugate for cell membrane: ex./em. at 495/519 nm) and *pseudo-blue* (DAPI for nuclei: ex./em. at 359/457 nm) are shown (two dyes in Figure 2c; three dyes staining in Figure 2e). Though the pseudo-red NHS dye can react with free amines present in the membrane of cancer cells, the α5β1 integrin (target) is a transmembrane protein. Binding of the F_n_M-coated NPs to that specific receptor (the PHSRN sequence of our F_n_M peptide is the synergy binding site, while GRGDSP is the primary recognition site of fibronectin to α5β integrins [49,50,51]) facilitates internalization; that could justify the augmented *pseudo-red* staining of breast cancer cells incubated with F_n_M-NPs over control (Figure 2d).

### 2.5. Quantification of mPEG-S-AuNP Contrast Agent in Solution and Ex Vivo Using a DE Micro-CT

**Figure 3:**
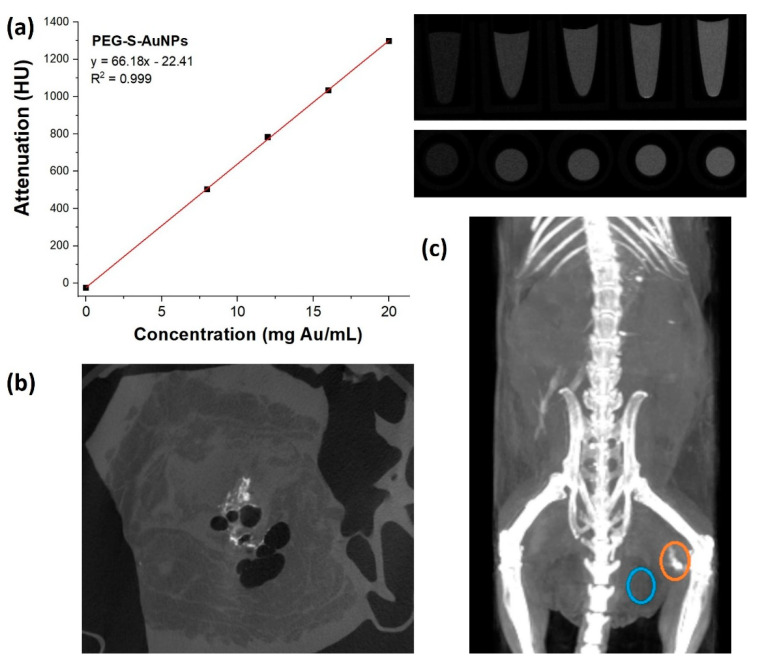
(**a**) Linear fitting of CT attenuation of mPEG-S-AuNPs in water between 0 and 20 mg Au/mL; top right: CT images of vials containing 0, 8, 12, 16, and 20 mg Au/mL; (**b**) CT scan of mPEG-S-AuNPs injected onto a pig intestine ex vivo at 16 mg Au/mL (black area denotes air pockets in the tissue; white area denotes contrast from NPs); (**c**) CT scan of a dead mouse injected with the same contrast material (30 μL; 0.48 mg Au). Orange-circled area shows the contrast material (bright), while blue-circled area represents tissue.

We first examined the CT attenuation of our mPEG-S-AuNPs contrast agent in solution. Homogenous aqueous dispersions were prepared between 0 and 20 mg Au/mL and scanned on a DE micro-CT at 50 keV. Results showed a linear correlation between CT signal and Au concentration (Figure 3a) and a high signal-to-solvent ratio. It is expected that a gradient in material concentration such as that of adherent/internalized (localized) NPs to cells would enhance the CT contrast vs. a homogenous NP dispersion, where the contrast is spread. A similar effect is anticipated when NPs are administered locally to tissue/tumor, which contains a significantly lower amount of water compared to an aqueous solution. To confirm, 30 μL of mPEG-S-AuNPs at 16 mg Au/mL (0.48 mg Au) were injected into a pig intestine (Figure 3b) as well as into the leg of a dead mouse (Figure 3c). Results showed pronounced CT contrast ex vivo with high separability in both experiments. In a mouse ex vivo, a signal-to-tissue ratio of 1.67 was achieved (Figure 3c; orange circle vs. blue: tissue), showing enhanced contrast in animal tissue as well. We envisioned that by functionalizing our Au nano-contrast agents with a peptide that targets specifically triple (−) breast cancer, early-stage breast cancer detection by CT can be achieved, as well as prolonged disease monitoring. Attaining that locally within tumors using small amounts of metal (to reduce toxicity and side effects), combined with DE-CT or PCD-CT, could lead to a breakthrough in CT imaging, nanotechnology, and breast cancer diagnosis.

### 2.6. Animal Studies of Au-Based Contrast Agents Using a Triple (−) Breast Cancer Model

**Figure 4:**
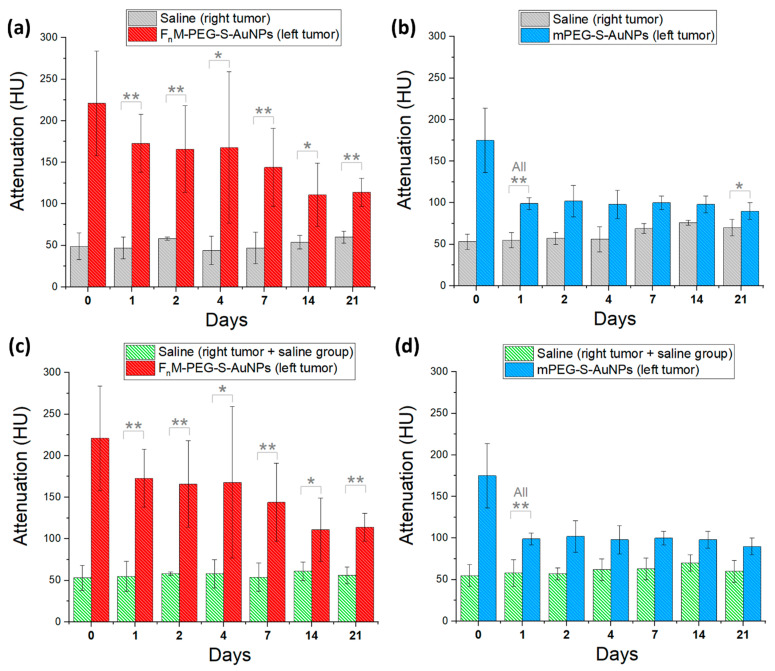
CT attenuation in breast tumor-bearing mice injected with (**a**) F_n_M-S-PEG-AuNPs contrast agent (left tumor; red) vs. saline (right tumor; gray), and (**b**) mPEG-S-AuNPs contrast agent (left tumor; blue) vs. saline (right tumor; gray). Figures (**c**) and (**d**) show the comparisons between F_n_M-S-PEG-AuNPs (left tumor) and mPEG-S-AuNPs, respectively, with the merged saline-treated group (left and right tumor of saline group + right tumor of x-PEG-S-AuNPs; green). Images were collected on days 0, 1, 2, 4, 7, 14, and 21. Statistical significances are given as follows: * *p* < 0.05 and ** *p* < 0.01.

**Figure 5:**
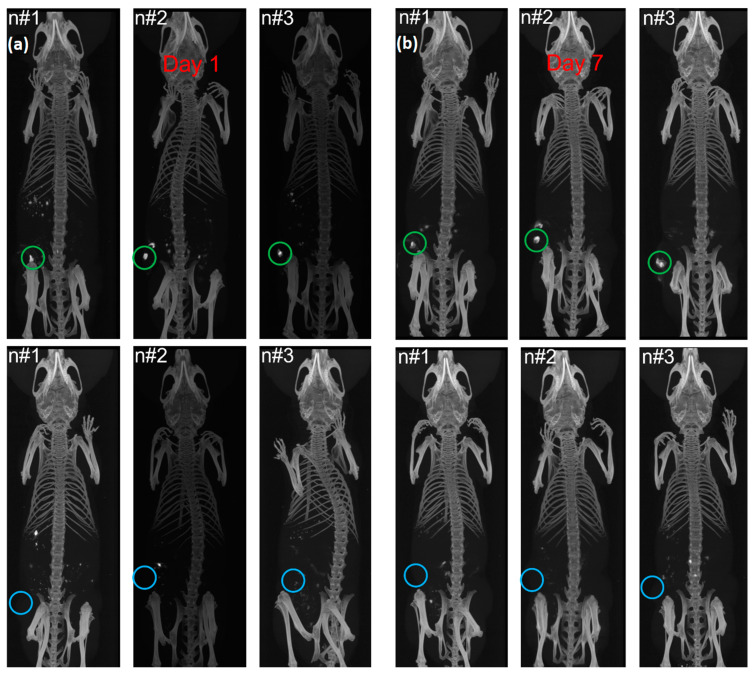
DE-CT coronal images of breast tumor-bearing mice injected with the F_n_M-S-PEG-AuNP contrast agent at 0.48 mg metal (Au in tumors is shown by green circle) and saline (blue circle) at (**a**) day 1 (left panels) and (**b**) day 7 (left panels). The tumors on animals’ right flank were injected with saline in all cases but are not delineated for simplicity. A DE-CT at 50 keV was used.

Focused on triple (−) breast cancer [46] that proliferates very aggressively and accounts for ~15% of all breast cancer cases, we engineered the ligand-specific KSSPHSRN(SG)_5_RGDSP-PEG-S-AuNPs X-ray contrast probe for long-term detection of triple (−) breast cancer in a mouse model. The F_n_M peptide has been used extensively by the group of Kokkoli for in vitro fluorescence detection of cancer cells [42,43]. DE-CT results showed significant signal enhancement for the F_n_M-PEG-S-AuNP probe vs. saline-treated mice (Figure 4 and Figure 5). The F_n_M-Au contrast agent was discernible on all days of the experiment and provided pronounced CT signal in tumors up to 21 days (last day of study due to tumor growth), though only 0.48 mg of Au (30 μL of 16 mg Au/mL sample) was administered. The CT signal remained concentrated (Figure 5) at the site of the tumor until day 14. After that, slight diffusion of AuNP-contrast within the tumor occurred, probably due to the increase of tumor size/volume (Figure S19). The X-ray signal enhancement at the site of tumor was statistically significant (*p* < 0.05) for the F_n_M-PEG-S-AuNP agent (left tumor) relative to the tumor injected with saline (right tumor) within the same animal(s) in all days (Figure 4a; *n* = 4). When we examined the animal group injected with F_n_M-PEG-S-AuNPs (left tumor; *n* = 4) relative to the saline group (left or right tumor; *n* = 3), statistical significance was achieved in days 0, 1, 2, 7, and 21 (0.00 < *p* < 0.02), but not in day 4 (*p* = 0.08) or 14 (*p* = 0.06). This was speculated to occur due to the smaller number of animals injected with saline (*n* = 3) vs. animals injected with the ligand-specific agent (*n* = 4). In support of that, by comparing the animals injected with F_n_M-PEG-S-AuNPs with the merged group of saline-injected tumors (left and right of the saline group + right tumor of peptide-PEG-S-AuNPs => *n* = 10), statistical significance was achieved in all days (*p* < 0.05; Figure 4c). This denotes that ligand-specific Au nanoprobes could yield a distinguishable X-ray attenuation from saline in breast tumors.

Similar correlations were done for mPEG-S-AuNPs. When comparing the non-specific mPEG-S-AuNPs (left tumor) with the saline-injected tumor (right tumor) within the same animal(s), statistical significance was achieved in all days (*p* < 0.05; Figure 4b). If we examine the animal group injected with mPEG-S-AuNPs (left tumor; *n* = 4) relative to the merged group of saline-injected tumors (left and right of the saline group + right tumor of mPEG-S-AuNPs => *n* = 10), statistical significance was achieved in all days as well (Figure 4d). Between the two Au-based contrast probes, the ligand-specific F_n_M-PEG-S-AuNP showed much higher CT contrast in all days, denoting specificity for breast cancer cells, similar to the fluorescent studies shown above (Figure 2c). Statistical comparison between the two, however, showed statistical significance and superiority of the F_n_M-PEG-S-AuNP probe over mPEG-S-AuNPs for days 1, 2 and 21 (*p* < 0.05). This is particularly important for long-term non-invasive imaging (using small amounts of metal), which is the aim of our study. We speculate that by increasing the number of animals per group, statistical significance can be achieved in all days as well.

On the other hand, iohexol administered locally to tumors (left; *n* = 4) at the same amount of metal (16 mg I/mL) showed minimal difference in CT attenuation compared to saline-treated tumors (right tumor; *n* = 4), with no statistical significance in all days (0.5 < *p* < 1.0, Figure S20a). The outcome was the same when we compared the iohexol-injected animals with the merged group of saline-injected tumors (left and right of the saline group + right tumor of iohexol => *n* = 10, Figure S20b), and implies that the small, iodinated contrast agents were cleared from the tumor vasculature due to size and non-specificity.

### 2.7. NP Biodistribution Studies/Contrast Retention and Accumulation in Tumors

**Figure 6:**
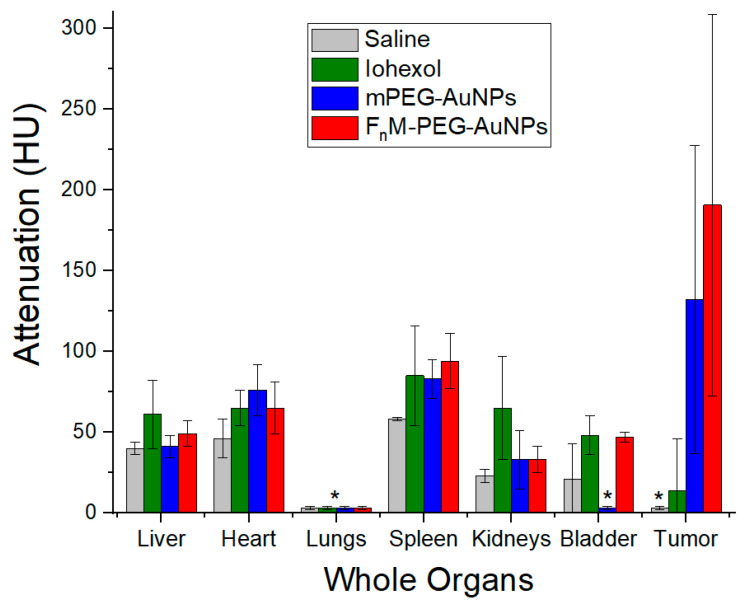
Whole organ biodistribution evaluated by DE-CT attenuation using the four different formulations: saline (gray), iohexol (green), mPEG-S-AuNPs (blue), and F_n_M-S-PEG-AuNPs (red). An *n* = 4 was used for all formulations, apart from for saline where *n* = 2 was used. Statistical significance (*p* < 0.05) was achieved between F_n_M-PEG-S-AuNPs vs. iohexol and saline within tumors. Asterisk denotes negative HU values due to air pockets within the organ.

Another set of animals was used to evaluate distribution of the administered contrast agents in different organs. To minimize the number of animals, we sacrificed mice with MDA-MB-231 tumors (grown for the same number of days as above) on day 14 post intra-tumoral administration of saline, iohexol, mPEG-S-AuNPs, and F_n_M-PEG-S-AuNPs, and measured their retention in excised organs by DE-CT relative to saline (Figure 6). Liver, kidneys, spleen, lungs, heart, bladder, and tumor were removed, weighed, and analyzed by DE-CT using a single spherical volume of interest (VOI) of a fixed size for each organ type. Statistical significance in organ weights was not shown (*p* > 0.05) in any of the study groups, denoting equality among organ measurements. Results showed minimal retention of iohexol in breast tumors after day 14 (green in Figure 6). The highest signal (relative to control) was found in kidneys, spleen, liver, heart, and bladder. The non-specific mPEG-S-AuNPs (in blue) showed retention in tumors (average Hounsfield Units: $\bar{\mathrm{HU}}$ = 132), with signal also found in spleen, kidneys, and heart. On the other hand, the α5β1 integrin-specific contrast agent F_n_M-S-PEG-AuNPs (in red) showed higher CT signal and retention in tumors after 14 days ($\bar{\mathrm{HU}}$ = 191), with NPs additionally found in spleen, bladder (denoting gradual clearance), and heart. Overall, at day 14, iohexol (standard; in green) and both NPs (blue and red) showed rather similar signal in liver, heart, lungs, spleen, and kidneys compared to saline (gray). This denotes that x-PEG-S-AuNPs do not accumulate in normal tissues and are in that respect similar to iohexol. However, tumors showed high accumulation and retention of ligand-specific NPs, with much higher and discernible signal relative to controls.

For tumors, 2–3 VOIs were used to (i) exclude bone encapsulated by tumor during growth in some animals (Figure S21a), and (ii) to quantify separately the localized metal contrast provided by NPs (Figure S21b). The averaged HU values obtained from the 2–3 different VOIs (white-striped colored bars), as well as the HU value acquired from the VOI with the highest signal/contrast (fully colored bars in Figure S21c) denote enhanced NP retention for the ligand-specific NP over the non-targeted NP. However, both NPs showed much higher and distinguishable contrast from saline and iohexol. The results were the same if median HU values were used instead (Figure S21d).

## 3. Materials and Methods

Materials. Dodecanethiol-functionalized gold nanoparticles were purchased from Nanoprobes Inc. Toluene, dichloromethane, hexane, ethanol, and tetraoctylammonium bromide (98%) were purchased from Fisher. Poly(ethylene glycol) 2-mercaptoethyl methyl ether (mPEG-SH, 5000 g/mol) and carboxymethyl-PEG-thiol (HOOC-PEG-SH, 5000 g/mol) were purchased from Laysan Bio Inc. The F_n_M peptide was purchased from Biomatik. NIH/3T3 mouse fibroblasts, DMEM, MDA-MB-231 human breast cancer cells, and L-15 media were acquired from ATCC. FBS, alamarBlue™ cell viability reagent, Alexa Fluor™ 594 NHS (succinimidyl ester) for staining peptide amino groups, wheat germ agglutinin-Alexa Fluor™ 488 conjugate for staining the cell membrane, and DAPI for staining nuclei, were purchased from ThermoFisher.

Synthesis of methyl-PEG-Gold Nanoparticles and HOOC-PEG-Gold Nanoparticles (x-PEG-S-AuNPs). PEGylated gold nanoparticles were prepared by ligand exchange of C12-S-AuNPs with excess mPEG-SH or HOOC-PEG-SH. Briefly, 1000 mg of x-PEG-SH (0.2 mmol, *M*_n_ = 5000 g/mol per manufacturer) were dissolved in 30 mL DCM and equilibrated at RT. Subsequently, a 20 mL DCM solution of C12-S-AuNPs (320 mg, 89% metal per TGA = 285 mg Au) was added dropwise (the polymer concentration was reduced to 2 wt%), followed by continuous stirring at RT for 18 h. The solvent was then evaporated, and the reaction mixture was precipitated in hexane to remove liberated dodecanethiol. The product was then dispersed in DI water at 10 wt% and centrifuged at 14,000 rpm for 2 h at 8 °C to remove unbound x-PEG-SH (supernatant), concentrating the nanocomposite precipitate (mPEG-S-AuNPs or HOOC-S-AuNPs). The final pellet was collected and dried under vacuum overnight at RT. Every step was monitored by TGA.

F_n_M Peptide Conjugation onto HOOC-PEG-S-AuNPs (F_n_M-PEG-S-AuNPs). The fibronectin-mimetic peptide KSSPHSRN(SG)_5_RGDSP was covalently conjugated to HOOC-PEG-S-AuNPs using EDC/NHS. Briefly, HOOC-PEG-S-AuNPs (160 mg; 26.4 wt% PEG from TGA => 42 mg PEG-COOH or 8.4 × 10^−6^ moles of -COOH) were dissolved in 4 mL water, vortexed, and sonicated until well-dispersed (~10 min). Subsequently, EDC (5.2 mg, 33.6 × 10^−6^ moles, 155.25 g/mol, 4 eq.) in 1 mL water was added, followed by NHS (3.9 mg, 33.6 × 10^−6^ moles, 115.09 g/mol, 4 eq.) in 1 mL water after 10 min, and the reaction was stirred for 2 h. After that, the F_n_M peptide H_2_N-KSSPHSRN(SG)_5_RGDSP-COOH (72.1 mg, 33.6 × 10^−6^ moles, 2145 g/mol, in 1.5 mL water, 4 eq.) was added dropwise to the above mixture and the reaction was left overnight at room temperature. The reaction was then dialyzed for 3 days against 4 L of DI water (with two changes per day) using a dialysis membrane (MWCO of 3.4 kDa, Fisher) to remove unreacted peptide, and finally the ligand-specific F_n_M-PEG-S-AuNPs were lyophilized to obtain a solid material.

Fibroblasts Studies/Viability. 3T3 mouse fibroblasts were initially grown at 37 °C in 5% CO_2_ in a tissue-culture treated flask in DMEM supplemented with 10% FBS and 0.5% antibiotics at a density of 1–2 × 10^5^ cells/mL (measured by trypan blue). mPEG-S-AuNPs were tested for biocompatibility indirectly by measuring the cell metabolic activity of 3T3 fibroblasts upon 24 h incubation/interaction with the NPs at 1:1 volume ratio (cell density was ~0.5–1 × 10^5^ cells/mL after dilution) at different NP concentrations: 0 μg/mL (control test), 10 μg/mL, 25 μg/mL, 50 μg/mL, and 100 μg/mL. PEG stabilizer was also examined. The alamarBlue assay was used for quantification of metabolic activity. Fluorescence measurements from resorufin in metabolically active cells (the non-toxic, cell-permeable compound resazurin is enzymatically reduced to the fluorescent reporter) were recorded on a Tecan Infinite M Nano plate reader at 530/590 nm (Ex./Em.) wavelengths. Three replicates per NP concentration were performed. The metabolic activity was recorded as a percentage (%) and referenced to 0 μg/mL NP concentration (control: 100% metabolic activity). Statistical analyses were performed using one-way ANOVA for comparison. Statistical significances are given as follows: * *p* < 0.05 and ** *p* < 0.01.

Breast Cancer Cell Studies/NP Binding. The x-PEG-S-AuNPs nanoprobes were also tested for binding with the human breast cancer cell line MDA-MB-231 (breast tissue; mammary gland). The cells were grown in a 25 cm^2^ tissue-culture treated flask in Leibovitz’s L-15 medium supplemented with 10% FBS at 37 °C in 100% air atmosphere for 3 days at a density of 2 × 10^5^ cells/mL. The cells were then diluted at 1:1 volume ratio with NP-supplemented medium at 250 μg Au/mL or control medium (the concentration was reduced to 1 × 10^5^ cells/mL), transferred into 25 cm^2^ treated culture flasks and incubated for 24 h under the same conditions. The medium was then aspirated, and the cells were washed with PBS three times to remove unbound NPs before optical microscopy images were taken.

Binding of the ligand specific probe towards α5β1 integrins of MDA-MB-231 cells was also examined by fluorescence confocal microscopy. Medium only and F_n_M-PEG-S-AuNPs were incubated with MDA-MB-231 breast cancer cells (at 2 × 10^5^ cells/mL density) at 1:1 ratio for 24 h at 250 μg Au/mL, washed twice with PBS, trypsinized, and fixed with 4% paraformaldehyde for 10 min at 1 mL cell suspension. The suspensions were then centrifuged at 125× *g* (~1100 rpm) for 5 min, the pellets were redispersed with 1 mL L-15 medium, and labeled with 5 µM Alexa Fluor™ 594 NHS (succinimidyl ester) at 37 °C for 60 min to stain the peptide (the amino groups specifically). Since free amines can be also found in cell membrane proteins, a control experiment (MDA-MB-231 cancer cells with medium only) was realized. The 1 mL suspensions were then centrifuged at 125× *g* (~1100 rpm) for 5 min to get a cell pellet, which was washed with L-15 medium. The 1 mL cell suspensions were then incubated with 5 µM wheat germ agglutinin-Alexa Fluor™ 488 conjugate (cell membrane staining dye) at 37 °C for 25 min, then centrifuged at 125× *g* (~1100 rpm) for 5 min to get a cell pellet, which was washed with 1 mL L-15 medium. The 1 mL cell suspensions were finally incubated with 2 µM DAPI (for staining the nuclei) at 37 °C for 10 min. Two cell pellet washings were performed, one with L-15 medium, and another one with PBS. The cell pellet was finally resuspended in 0.3 mL PBS to concentrate cells, and a drop of each stained suspension was added on a glass slide, followed by cover slip mounting with nail polish. Fluorescence detection was measured on a confocal laser scanning microscope (Leica SP8) at different channels.

Characterization. SEC of mPEG-SH and HOOC-PEG-SH (at 5 mg/mL) was performed using THF as the eluent solvent (HPLC grade) with a flow rate of 1 mL/min at 25 °C, and the number-average molecular weight (*M_n_*) and dispersity (*Đ*) were measured. A Waters 717 Plus autosampler was used, equipped with a 515 HPLC pump, a 2410 differential refractometer, and 5 Styragel HR GPC columns connected in the following order: 500, 103, 104, 105, and 100 Å, which were calibrated against seven poly(styrene) standard samples. The results were processed by the Empower 3 software (Waters). For TGA analysis, the samples were vacuum-dried overnight at room temperature before the analysis, and measured (in triplicate) on a TA Instrument Discovery thermal analyzer (New Castle, DE, USA) at a heating rate of 20 °C/min under N_2_, from 50–600 °C. UV–Vis images were collected on a Thermo Scientific Evolution 60 s between 300–800 nm. The hydrodynamic size was measured by DLS at 25 °C on a Malvern Zetasizer Nano ZS90 with a He/Ne laser (633 nm) at 173° collecting optics. x-PEG-stabilized AuNP dispersions were prepared at 0.2 mg/mL using DI water (filtered with a 0.2 μm syringe filter). Poly(styrene) spheres of 100 nm were used as a positive standard and 0.2 μm filtered DI water as a negative control (~0 counts). Data were analyzed by Malvern Dispersion Technology Software 4.20. For amino acid analysis, the F_n_M peptide (positive control) and unknown samples were hydrolyzed in 6 N HCl at 105 °C for 24 h, the solvent was evaporated, and samples were dissolved in 1 mL DI water. In the case of NPs, a centrifugation step was performed post water addition and the supernatant was collected. Quantification was performed based on calibration curves of the amino acids Ser, Gly, and Pro between 0 and 50 μM. For NP peptide quantification, 6 mg of mPEG-S-AuNPs and F_n_M-PEG-S-AuNPs were used in 1 mL of water. Samples were run on a Shimadzu 8040 Triple Quadrupole LC/MS; 30 μL of unknown sample were injected on a Thermo Fisher ODS C18 Hypersil 4.6 × 60 mm column (3 μm particle size), which was kept at 40 °C temperature. The gradient mobile phase consisted of 0.1 M formic acid in water (solvent A) and 0.1% formic acid in acetonitrile (solvent B), started with 95:5 A/B, increased over 2 min to 100% acetonitrile, and held at 100% acetonitrile for 2 min, pumped at a 0.5 mL/min flow rate. The total analysis run was set to 5 min for all the samples. Two methanol washings were carried out after each measurement. TEM images were collected on a Philips CM12 electron microscope operated with an acceleration voltage of 120 kV. C12-S-AuNPs were prepared at 0.5 mg/mL in hexane, while mPEG-S-AuNPs, HOOC-PEG-S-AuNPs, and F_n_M-PEG-S-AuNPs were prepared in water at 0.5 mg/mL. To estimate core size, more than 300 NPs were measured and analyzed with ImageJ. Fluorescence confocal images were collected on a confocal laser scanning microscope Leica SP8 at different channels.

Animal Studies. The MDA-MB-231 breast cancer cell line xenograft model in female mice was used. All procedures were in accordance with the UMass Chan Medical School IACUC protocol. Mice were Fox Chase SCID, strain code: 236 (CB17/Icr-Prkdc scid/IcrIcoCrI), 5–7 weeks old, female, from Charles River Laboratories. Mice were injected subcutaneously with 4 × 10^6^ cells on both left and right lower flanks. Study agents were delivered when tumors were ~200–250 mm^3^ (about 18 days). Tumor size was measured by calipers over the course of study. Animals were randomly distributed into 4 study groups: (a) saline (*n* = 3), (b) iohexol (*n* = 4), (c) mPEG-S-AuNPs (*n* = 4), and (d) F_n_M-PEG-S-AuNPs (*n* = 4). Study agents were delivered as a single 30 μL intra-tumoral injection with 16 mg metal/mL (~0.48 mg metal) in the left tumor, with saline injected in the right tumor, apart from the saline group where both tumors were injected with saline. CT images were acquired on days 0 (immediately after administration), 1, 2, 4, 7, 14, and 21 to determine tumor retention, clearance, and NP organ distribution. For CT imaging, mice were under isoflurane anesthesia (delivered by nose cone). A DE micro-CT (MILabs VECTor^6^ CT^UHR^) was used at the following scanning parameters: 50 keV, 0.21 mA current, 70 sec exposure time, total image time 3–6 min, while all images were set at the same color scale, gray <−1000, 3000>. For tumor CT quantification in animals (attenuation expressed in HU), spherical VOIs were taken in left and right tumors and quantified in excel.

Another set of animals (*n* = 4; apart from saline where *n* = 2) was used to evaluate distribution of the administered contrast agents in different organs at day 14. For each organ, quantification was performed by DE-CT using a single spherical VOI. For tumors on the other hand, 2–3 different spherical VOIs were used since growing tumors in some cases also contained bone (Figure S21). In tumors with NPs, quantification was measured by averaging the HU values obtained from the 2–3 different VOIs, as well as by measuring the spherical VOI with the highest signal/contrast (Figure 6 and Figure S21). To account for discrepancies in measurements, both average HU values with error bars and median HU values are shown.

## 4. Conclusions

Increasing the accumulation of AuNPs in tumors has been the object of study for many years. The general strategy so far has been focused on iv administration of NPs, with the aim of a small amount to accumulate in the tumor (similar to therapeutic injectable materials) and offer diagnosis, with engineering strategies ranging from modifying the NP surface with ligands (targeting specificity; *active tumor targeting*) or varying the NP size (*passive targeting mechanism*). Our approach combines localization of metal contrast through molecular specificity for breast cancer cells receptors (overexpressed α5β1 integrins) with spectral CT, after intra-tumoral administration, to achieve long-term detection of breast cancer. Unlike published data where 5–54 mg Au [23,24,29,31,32,36,38,40] have been utilized for CT detection in mice, we only used 0.5 mg Au and clearly detected signal in tumors for up to 21 days. Since the 10-year fatality rate of breast cancer patients who were assisted from early detection has been significantly decreased in the last years, our NP-enhanced CT diagnosis could lead to significant advances in molecular sciences, nanomaterials chemistry, radiology, and medicine. In future studies, our NP technology will be coupled with thermal ablation, radiation therapy, or engineered to carry chemo/immuno-therapeutics for CT theranostic probes.

## Supplementary Materials

The following supporting information can be downloaded at: https://www.mdpi.com/article/10.3390/ijms23179926/s1.
