# Supplementary material for: Ligand-Specific Nano-Contrast Agents Promote Enhanced Breast Cancer CT Detection at 0.5 mg Au
Source: Int J Mol Sci. 2022 Sep 1;23(17):9926. doi: 10.3390/ijms23179926 (PMC9456125; doi:10.3390/ijms23179926)
Supplement: Supplementary file 1 [file ijms-23-09926-s001.zip › ijms-1882511-supplementary.pdf]

Supporting Information

Ligand-Specific Nano-Contrast Agents Promote Enhanced Breast Cancer CT Detection at 0.5 mg Au

Kalyan Ramesh,[∇] *Alice Truong,*[#] *Yuzhen Wang,*[#] *Mary Rusckowski,*[*,#] *Manos Gkikas*[*,∇]

[∇] University of Massachusetts Lowell, Department of Chemistry, Lowell, Massachusetts 01854, U.S.A.

[#] University of Massachusetts Chan Medical School, Department of Radiology, Worcester, Massachusetts 01655, U.S.A.

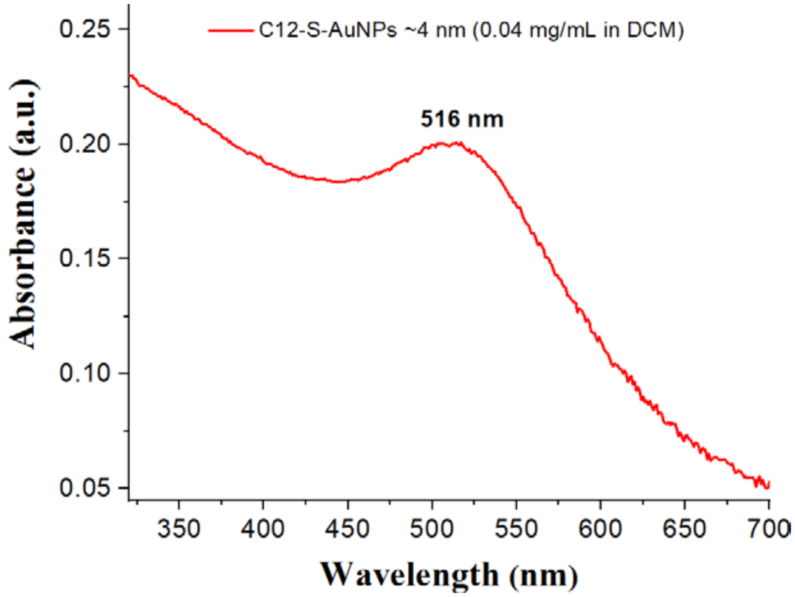

Figure S1. UV-Vis results of ~3.7 nm C12-S-AuNPs at dichloromethane at 0.04 mg/mL.

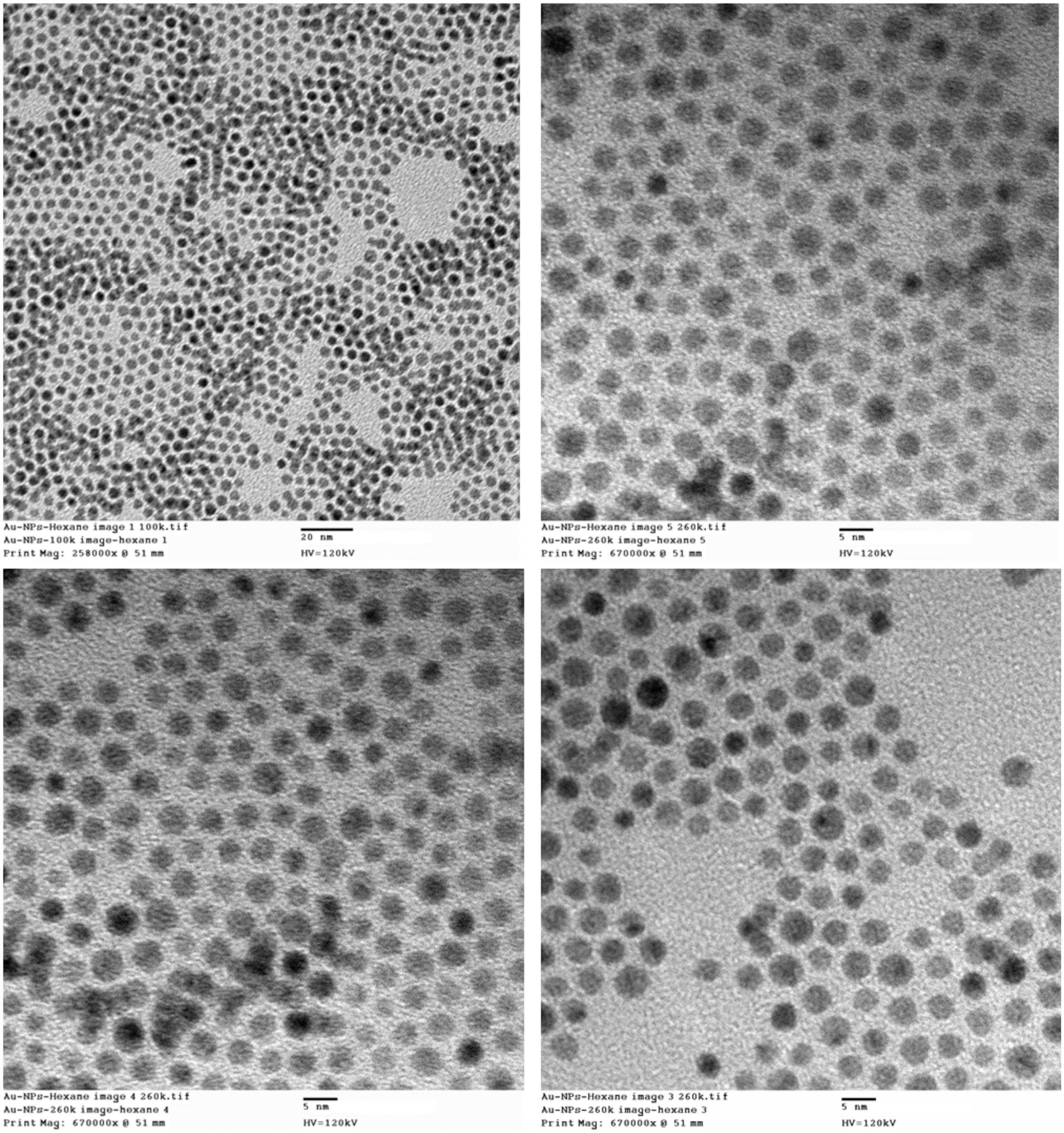

Figure S2. TEM results of ~3.7 nm **C12-S-AuNPs** (0.5 mg/mL in hexane).

TGA of C12-S-AuNPs (Table for Figure S3).

**Sample**	**Decomp. T1 (^{o}C)**	**Weight Loss 1 (%)**	**Decomp. T2 (^{o}C)**	**Weight Loss 2 (%)**
	249.78	11.63	-	-
	248.86	11.09		
	249.70	11.50		
Average	**249.5 ± 0.5**	**11.4 ± 0.2**	-	-

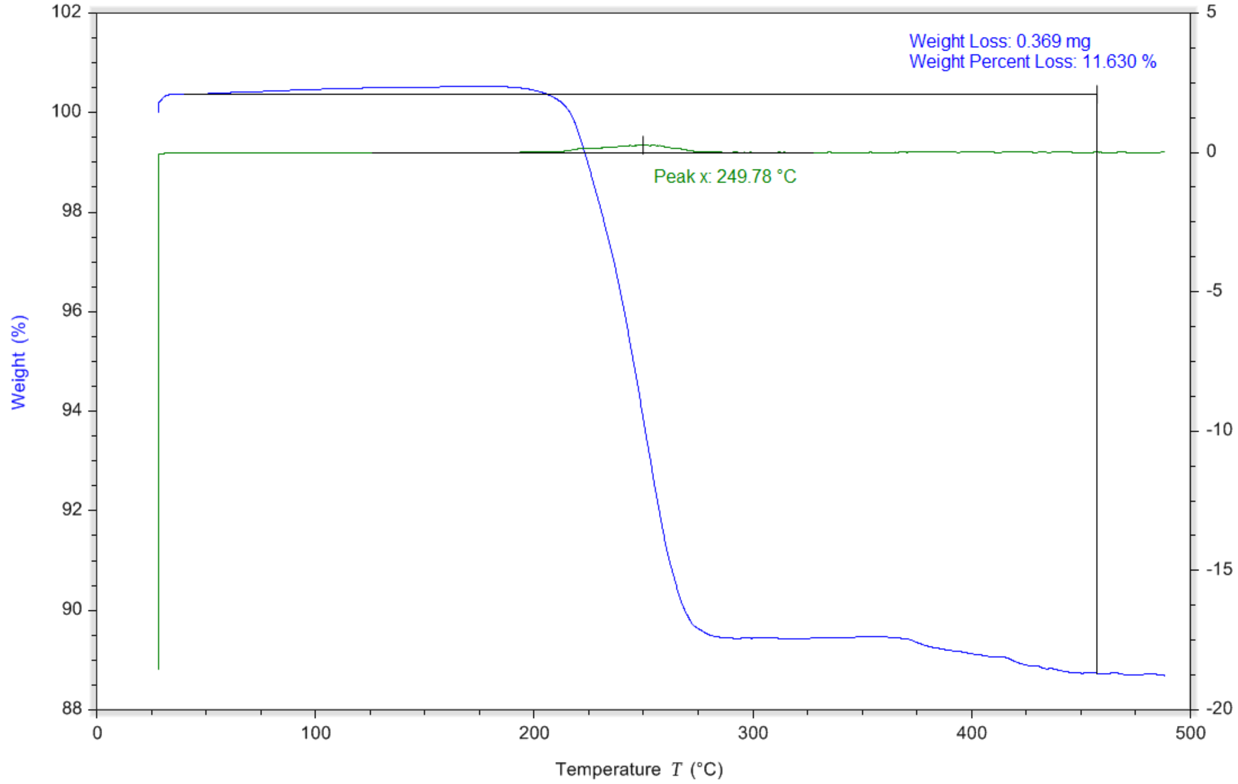

C12-S-AuNPs #1

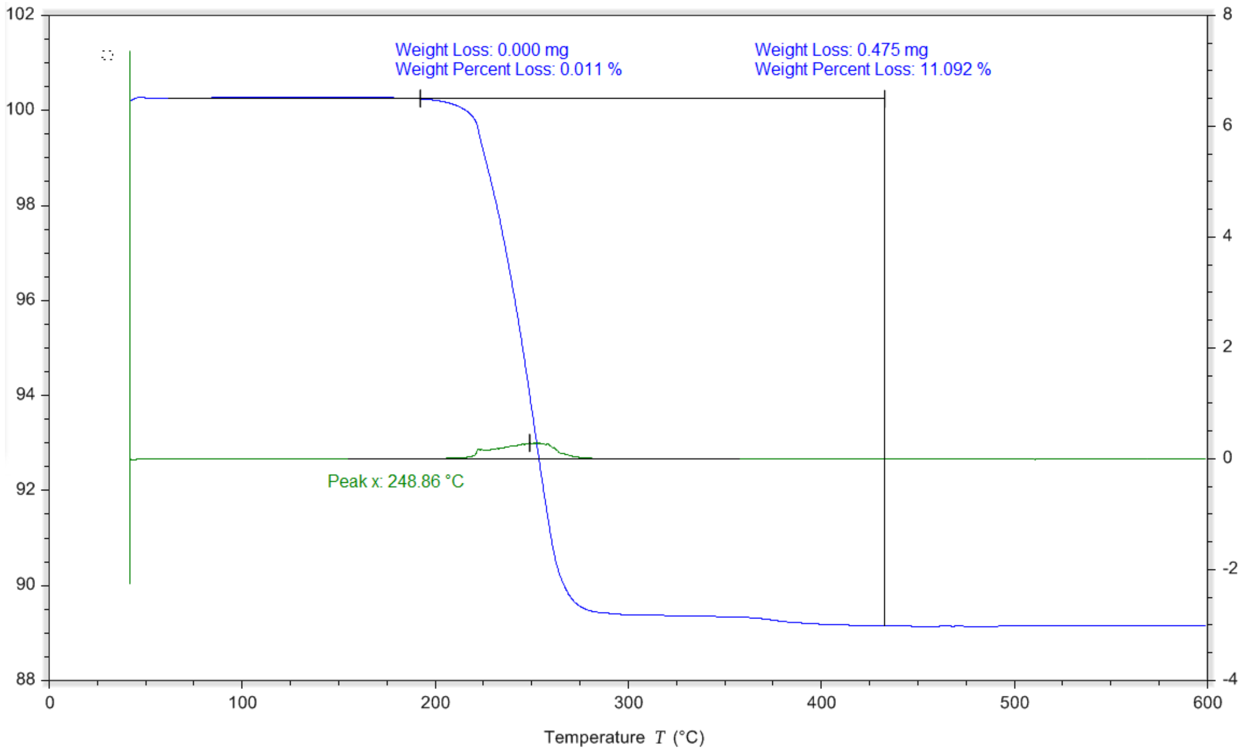

C12-S-AuNPs #2

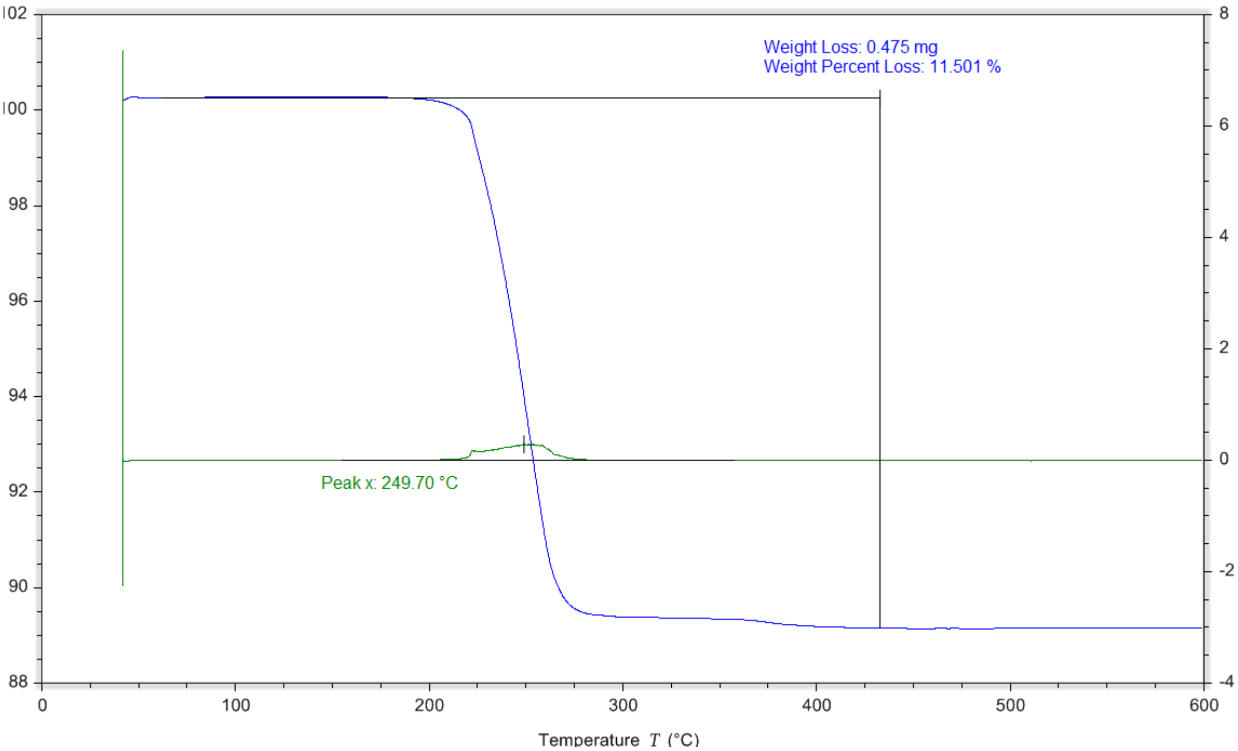

C12-S-AuNPs #3

Figure S3. TGA results of C12-S-AuNPs.

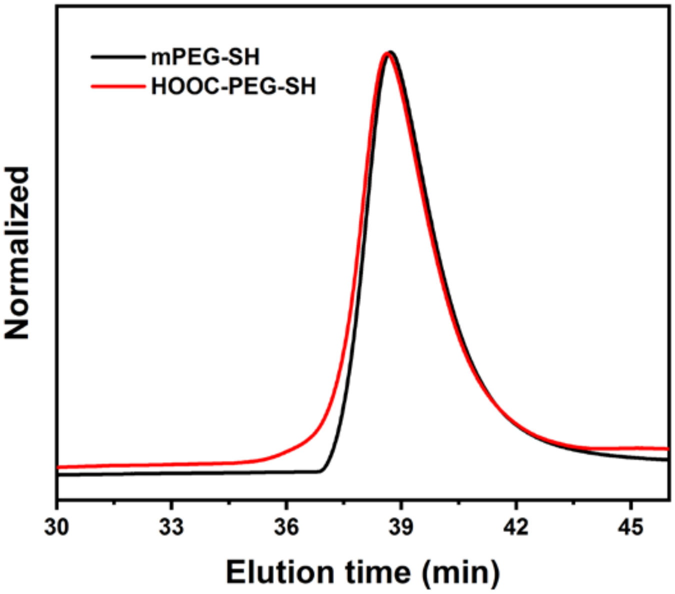

Figure S4. SEC traces of mPEG-SH and HOOC-PEG-SH in THF.

TGA of mPEG-S-AuNPs (Table for Figure S5).

**Sample**	**Decomp. T1 (ºC)**	**Weight Loss 1 (%)**	**Decomp. T2 (ºC)**	**Weight Loss 2 (%)**
	247.04	8.91	397.10	30.64
	248.97	8.98	395.86	29.88
	245.67	8.41	395.39	32.57
Average	**247.2** ± **1.7**	**9.0** ± **0.1**	**396.1** ± **0.9**	**31.0** ± **1.4**

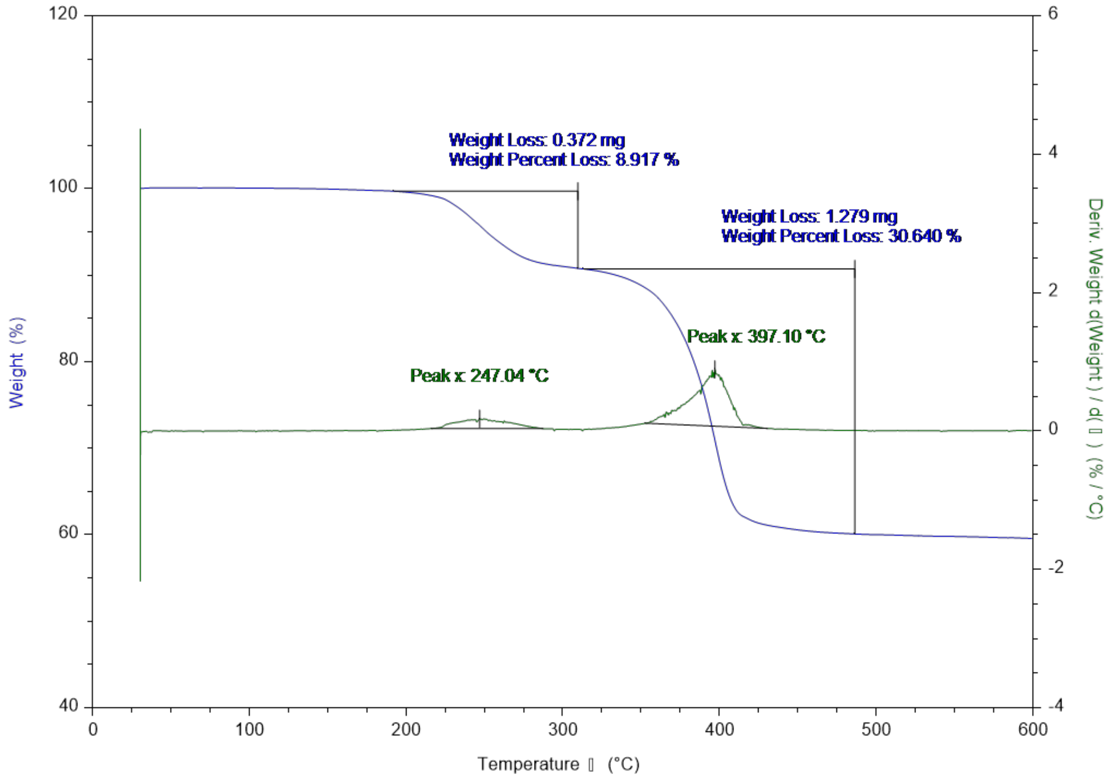

mPEG-S-AuNPs #1

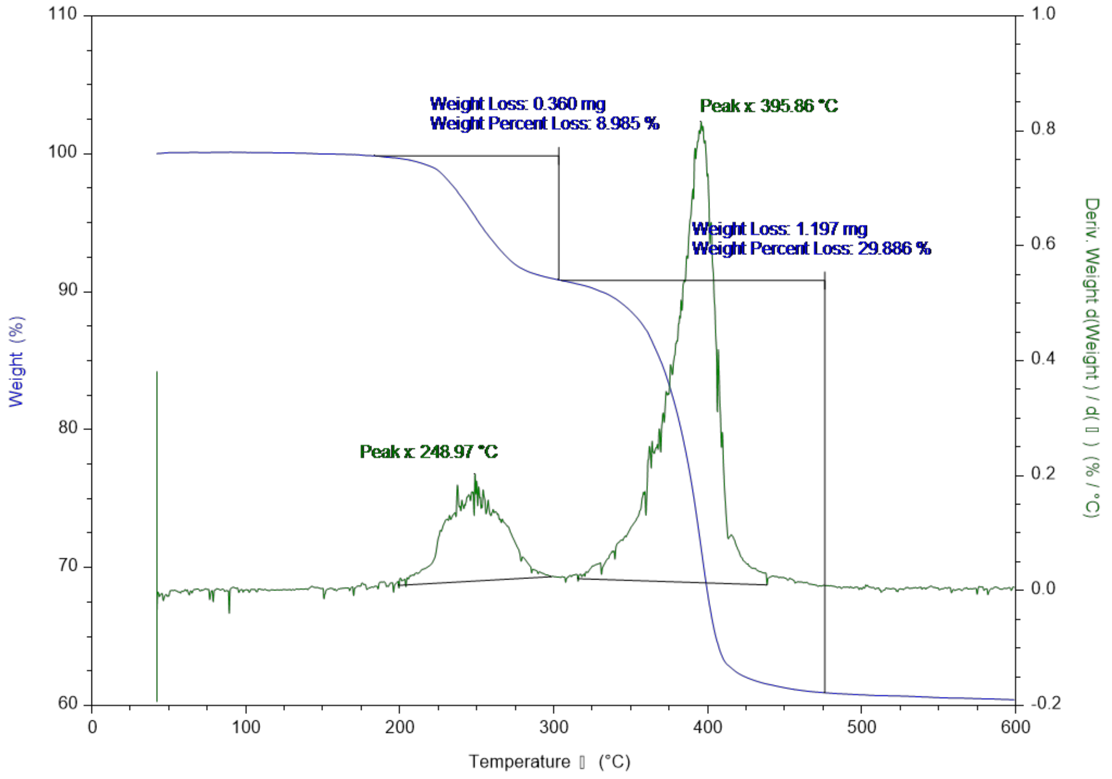

mPEG-S-AuNPs #2

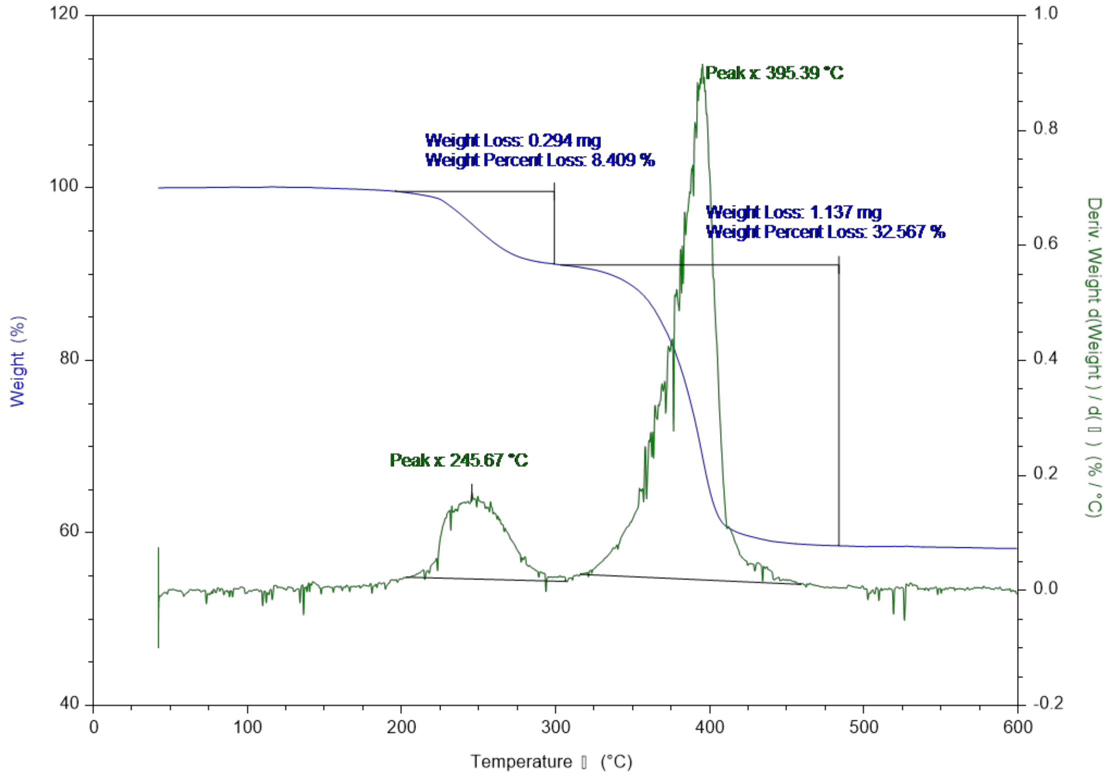

mPEG-S-AuNPs #3

Figure S5. TGA results of synthesized mPEG-S-AuNPs.

TGA of mPEG-SH (Table for Figure S6).

**Sample**	**Decomp. T1 (ºC)**	**Weight Loss 1 (%)**	**Decomp. T2 (ºC)**	**Weight Loss 2 (%)**
	-	-	403.20	99.32
	-	-	405.42	99.00
	-	-	402.58	98.83
Average	**-**	**-**	**403.7** ± **1.5**	99.1 ± **0.3**

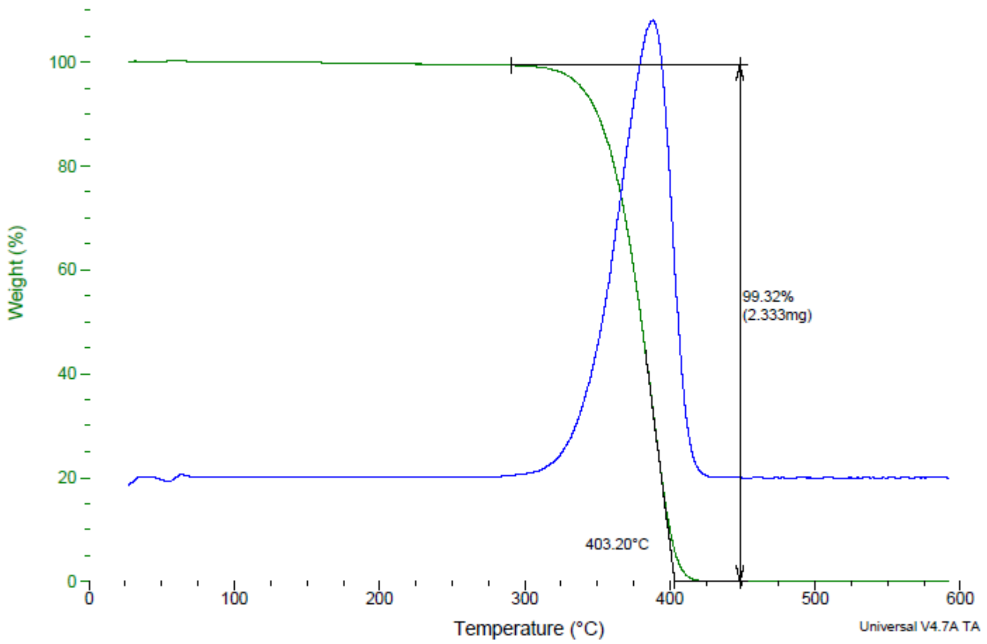

mPEG-SH #1

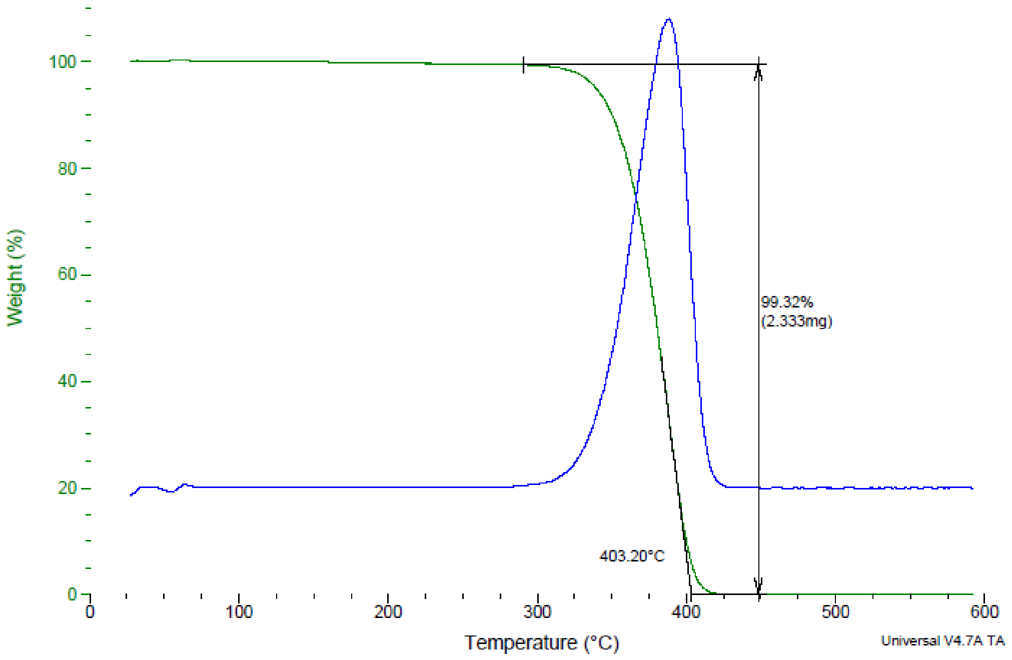

mPEG-SH #2

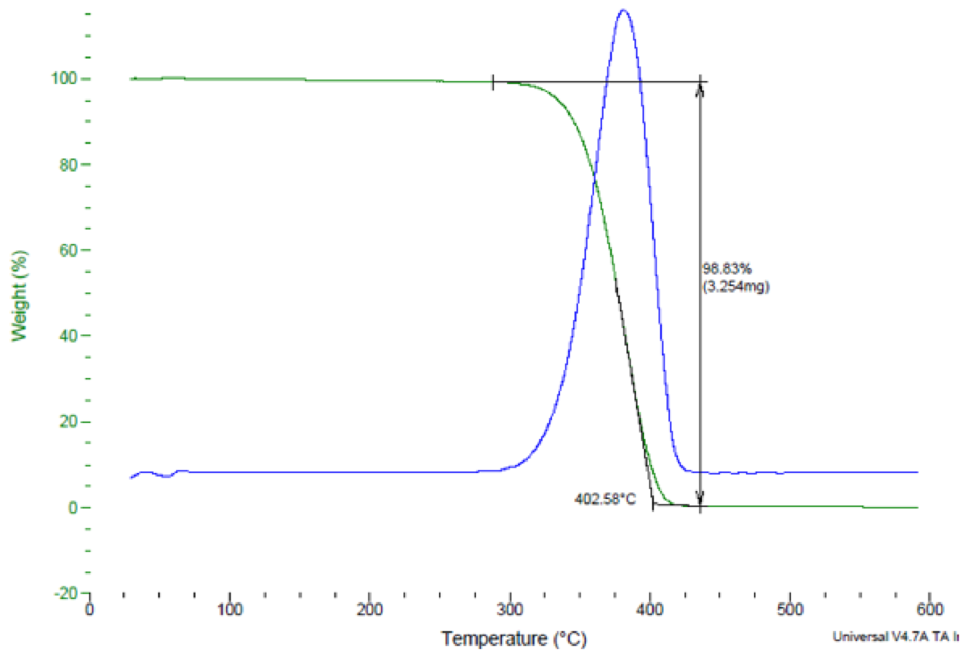

mPEG-SH #3

Figure S6. TGA results of mPEG-SH.

TGA of HOOC-PEG-S-AuNPs (Table for Figure S7).

**Sample**	**Decomp. T1 (ºC)**	**Weight Loss 1 (%)**	**Decomp. T2 (ºC)**	**Weight Loss 2 (%)**
	259.10	12.27	400.98	26.18
	254.47	12.86	402.84	26.67
	260.66	12.49	405.70	26.40
Average	**258.1** ± **3.2**	**12.5** ± **0.3**	**403.2** ± **2.4**	**26.4** ± **0.3**

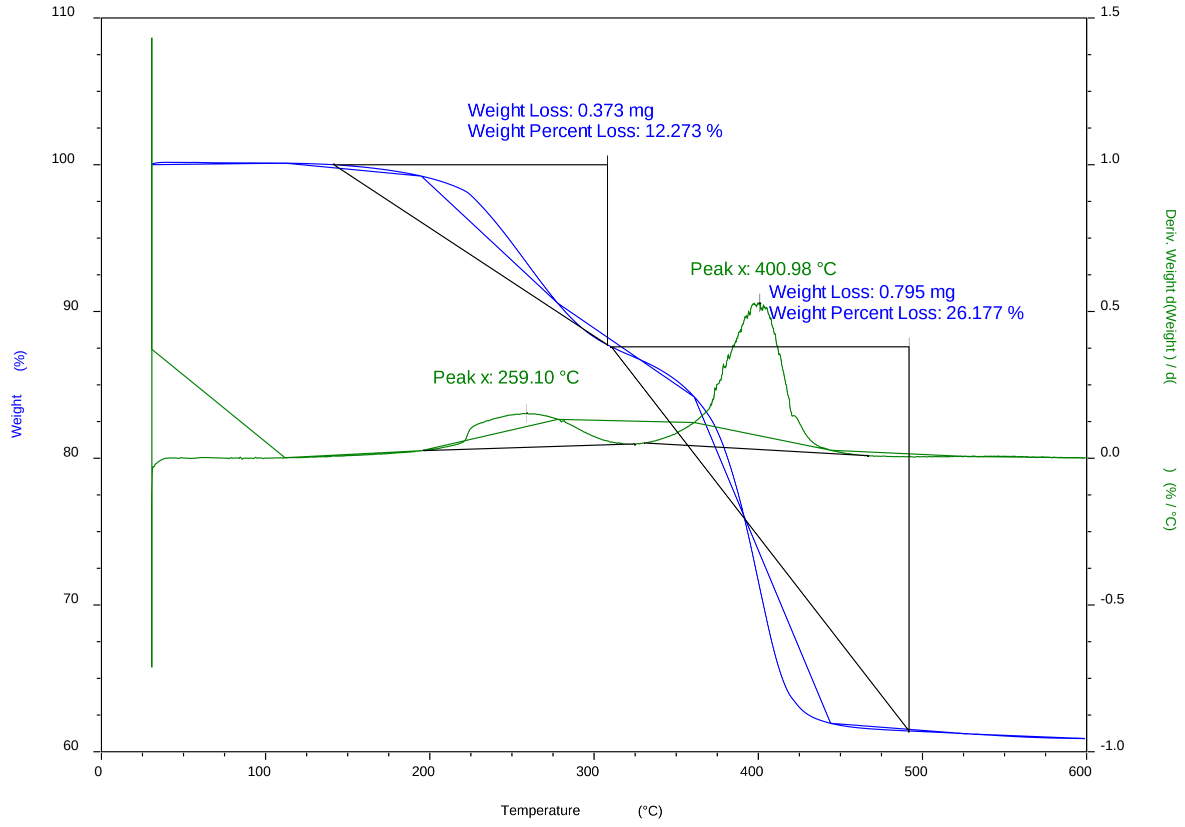

HOOC-PEG-S-AuNPs #1

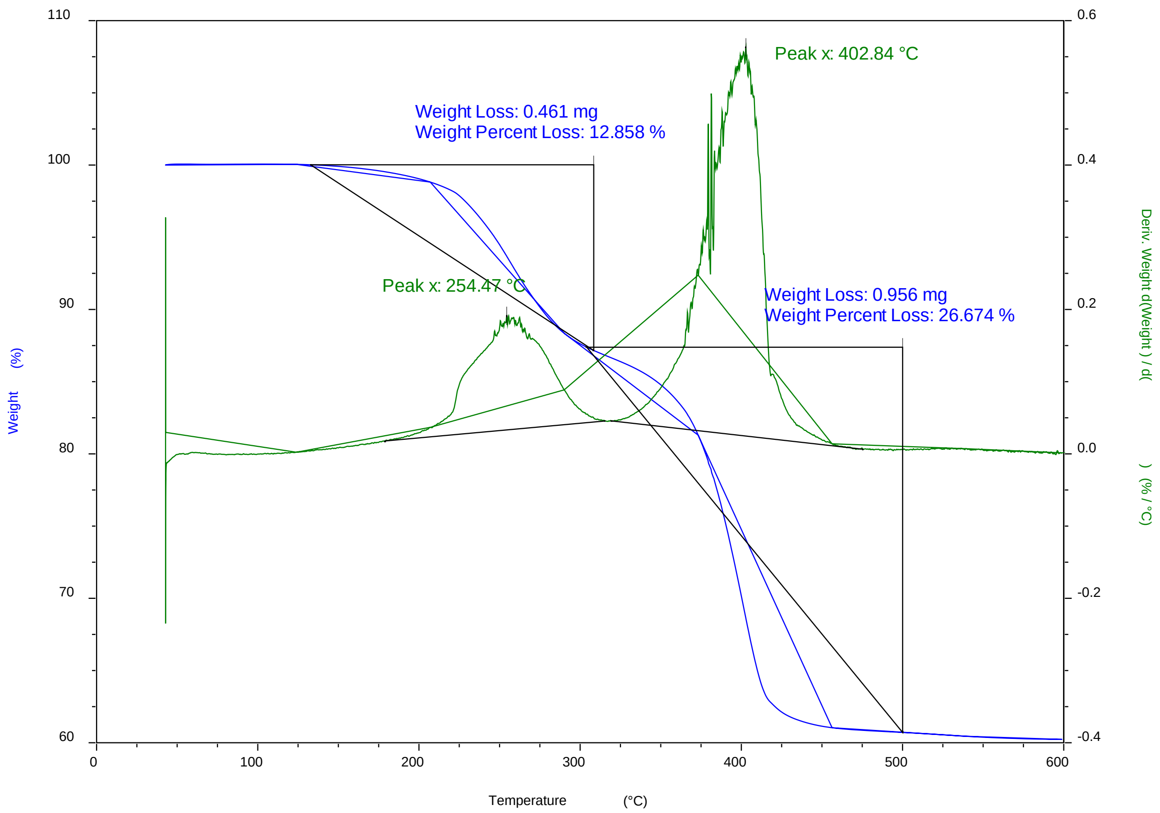

HOOC-PEG-S-AuNPs #2

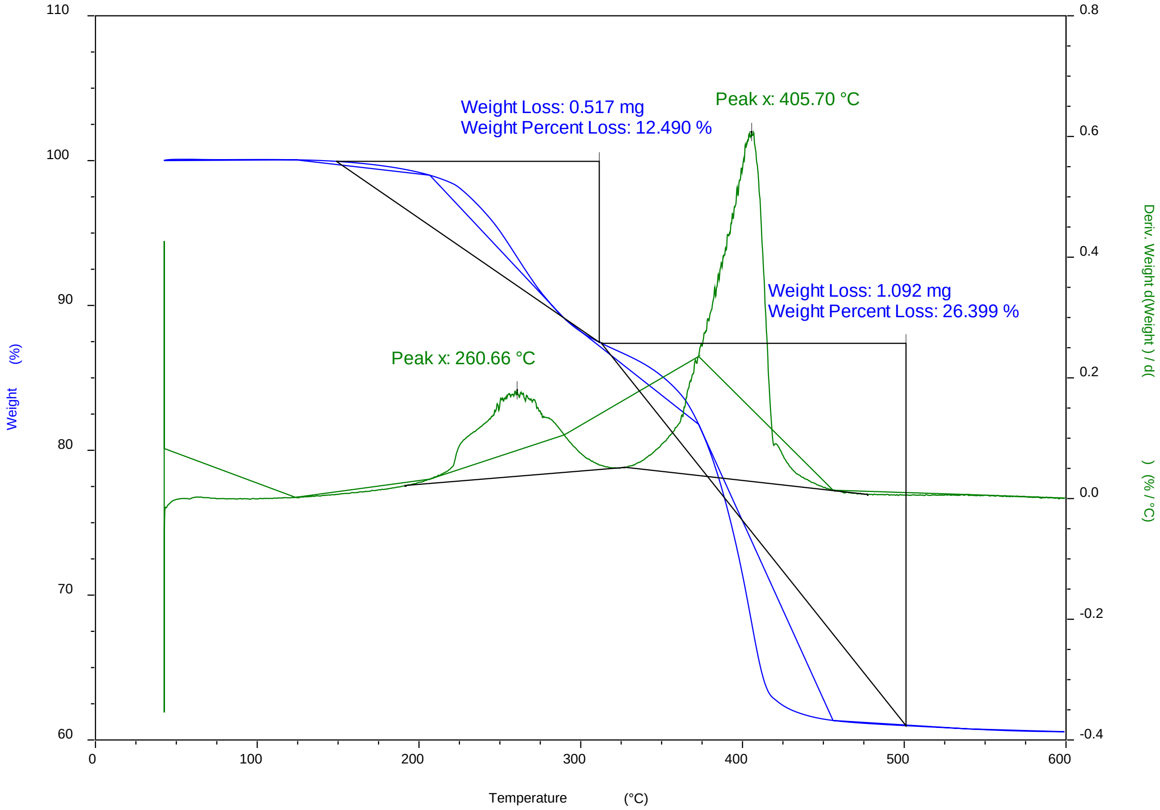

HOOC-PEG-S-AuNPs #3

Figure S7. TGA results of synthesized HOOC-PEG-S-AuNPs.

TGA of HOOC-PEG-SH (Table for Figure S8).

**Sample**	**Decomp. T1 (ºC)**	**Weight Loss 1 (%)**	**Decomp. T2 (ºC)**	**Weight Loss 2 (%)**
	-	-	405.81	98.06
	-	-	406.53	98.02
	-	-	402.59	98.36
Average	**-**	**-**	**405.0 ± 2.1**	**98.15 ± 0.19**

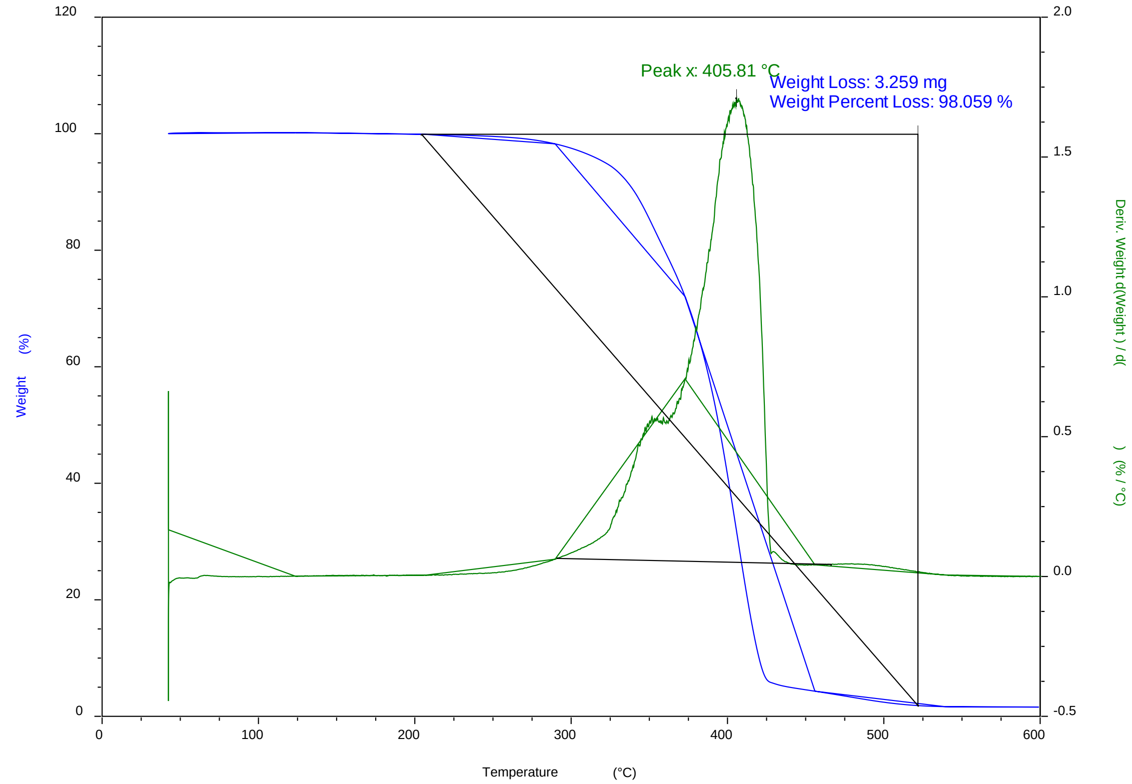

HOOC-PEG-SH #1

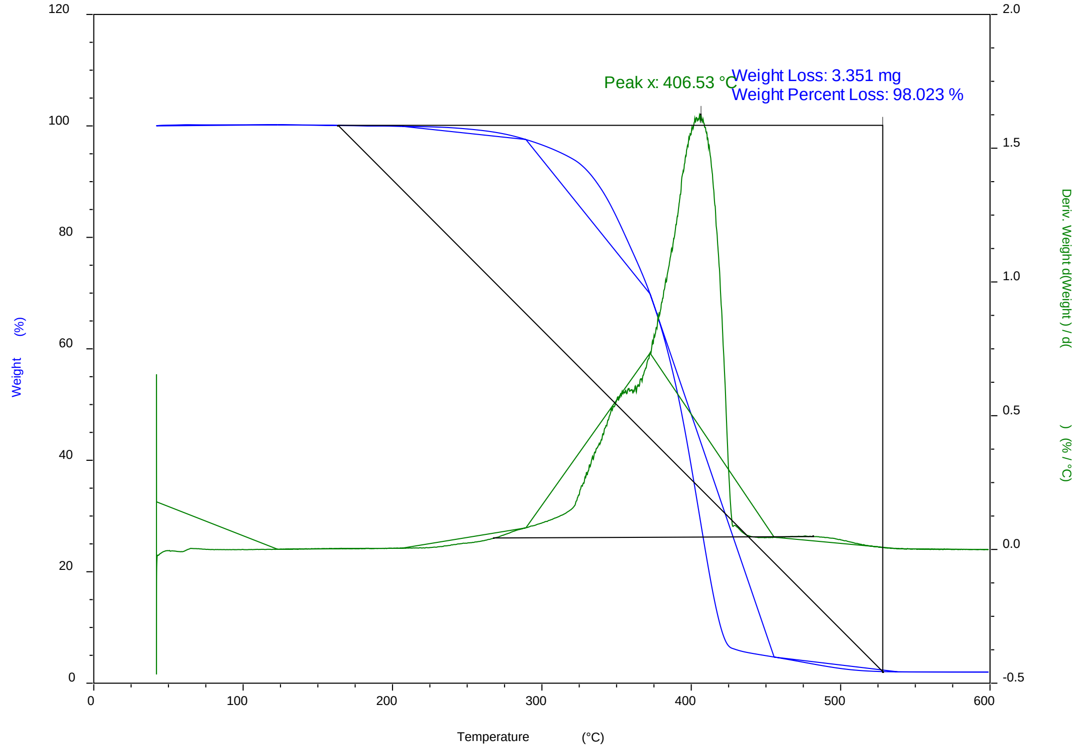

HOOC-PEG-SH #2

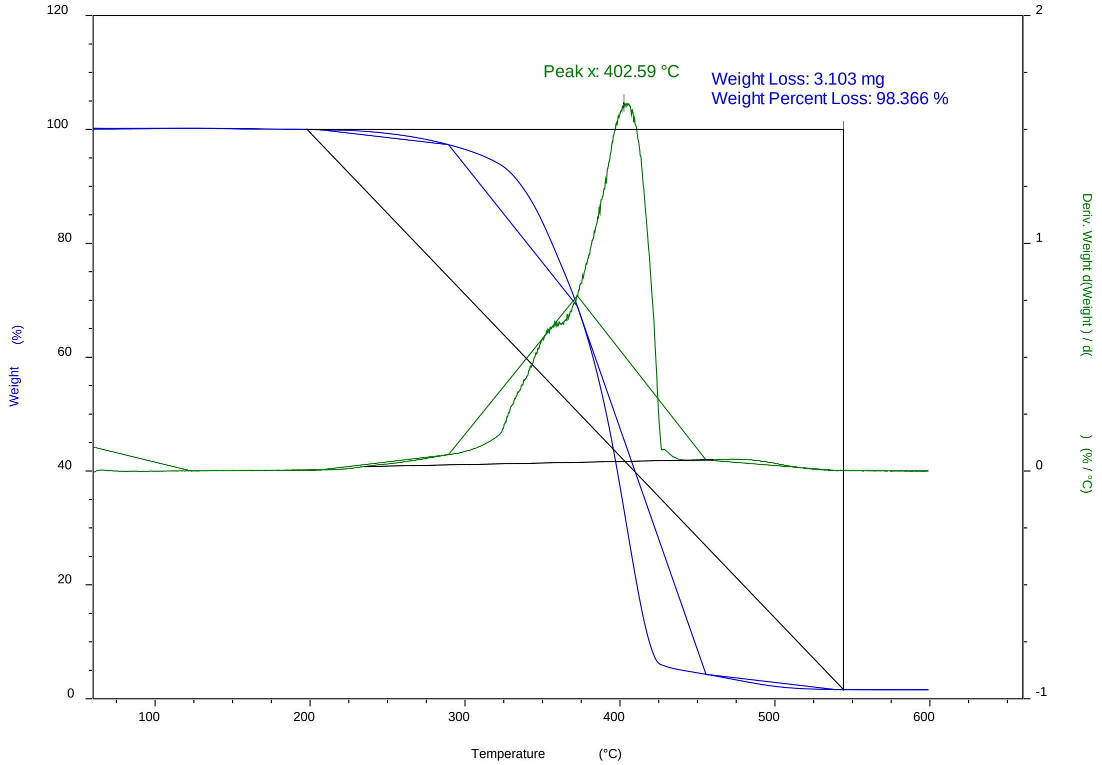

HOOC-PEG-SH #3

Figure S8. TGA results of HOOC-PEG-SH.

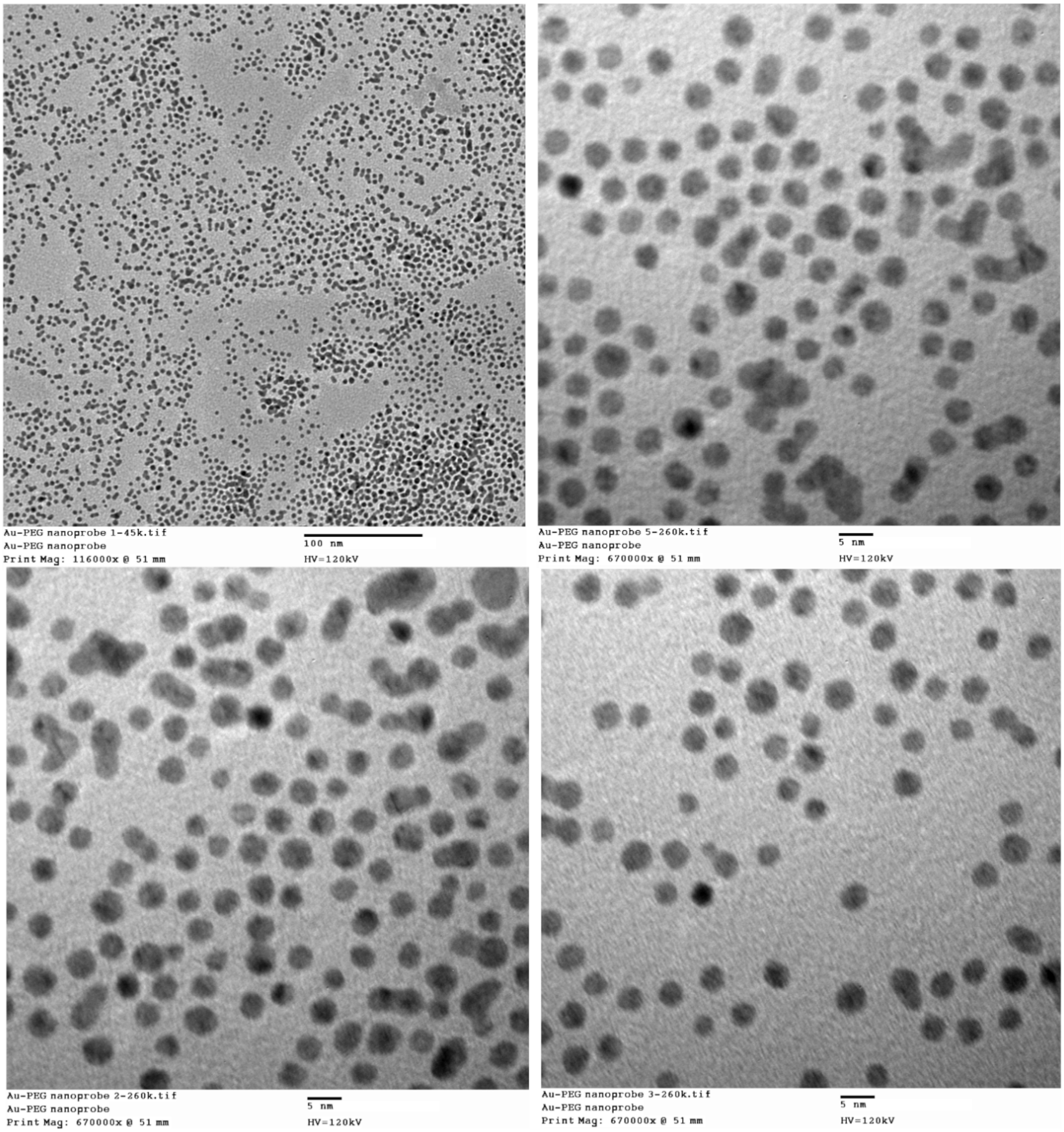

Figure S9. TEM results of ~3.4 nm **mPEG-S-AuNPs** (0.5 mg/mL in water).

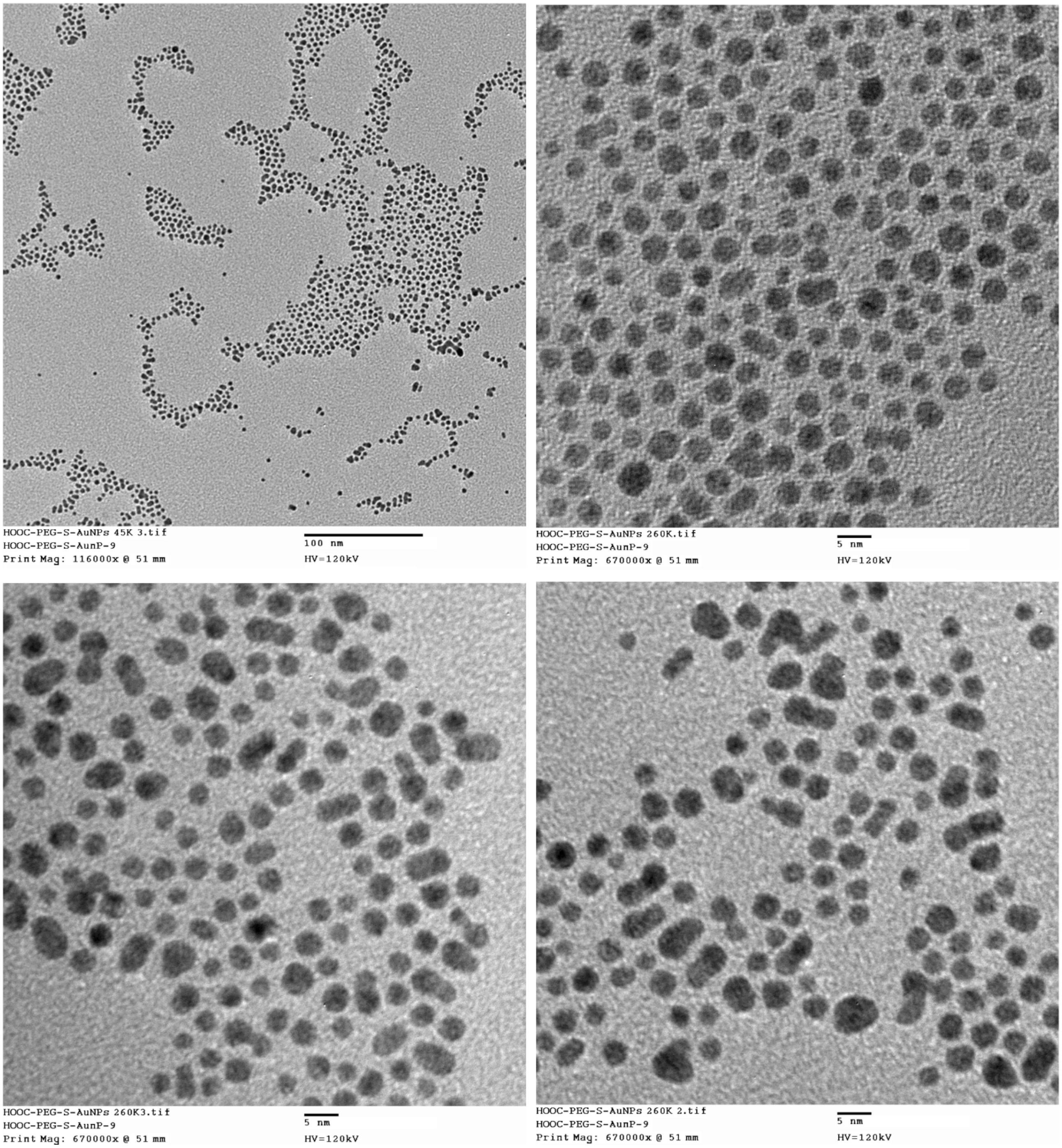

Figure S10. TEM results of ~3.1 nm **HOOC-PEG-S-AuNPs** (0.5 mg/mL in water).

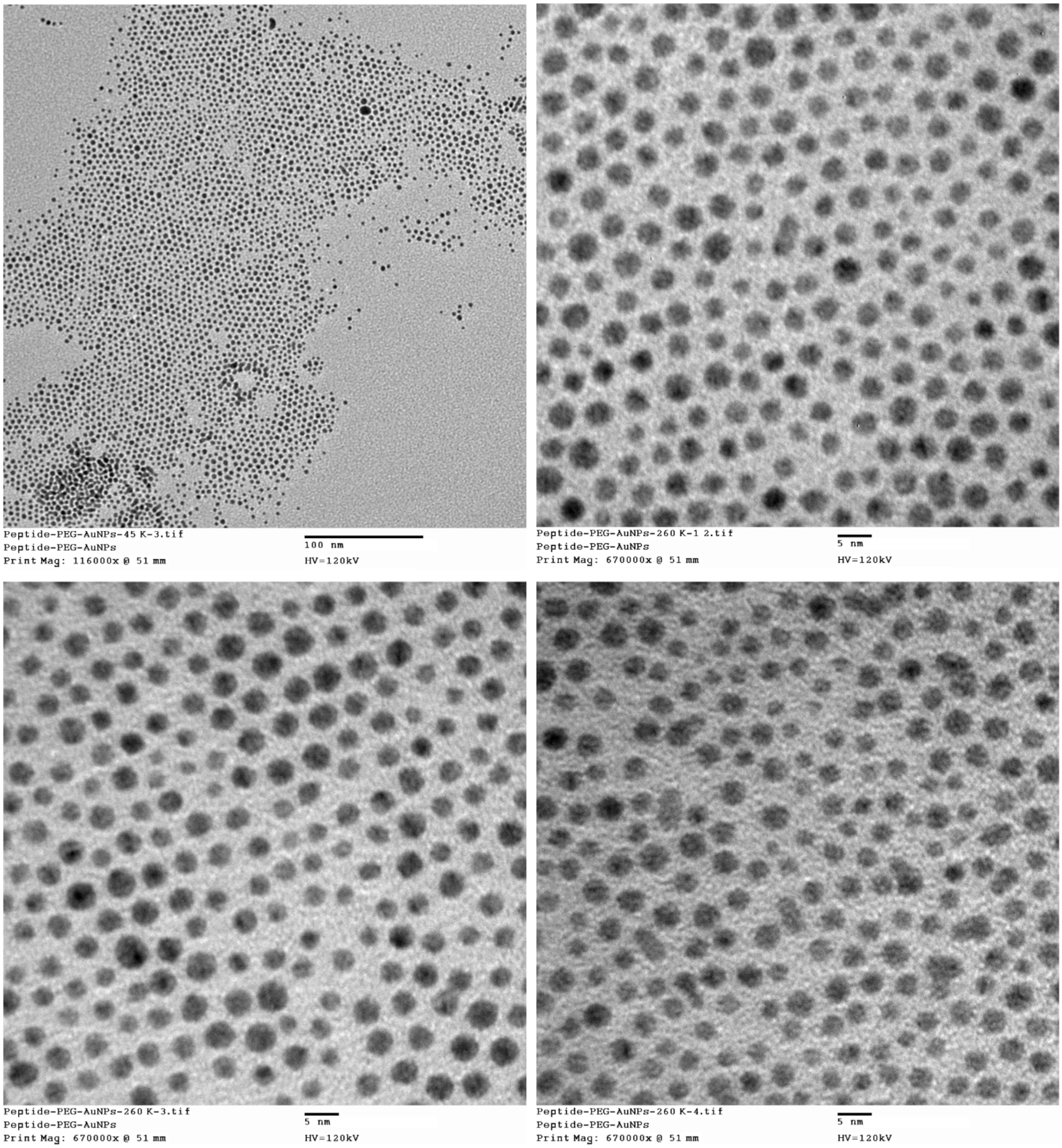

Figure S11. TEM results of ~3.1 nm **F_nM-PEG-S-AuNPs** (0.5 mg/mL in water).

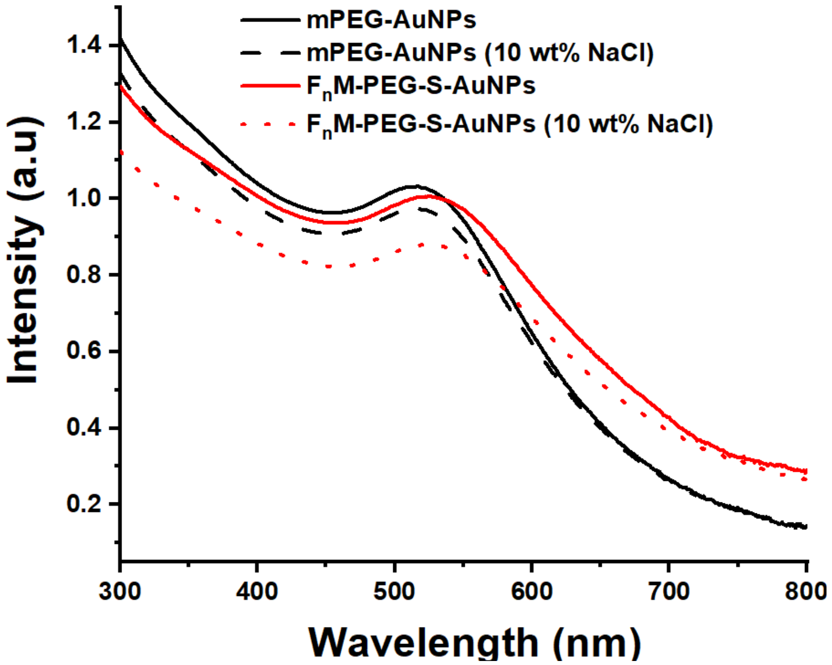

Figure S12. UV-Vis results of mPEG-S-AuNPs and F_nM-PEG-S-AuNPs in 10% NaCl at 0.25 mg/mL.

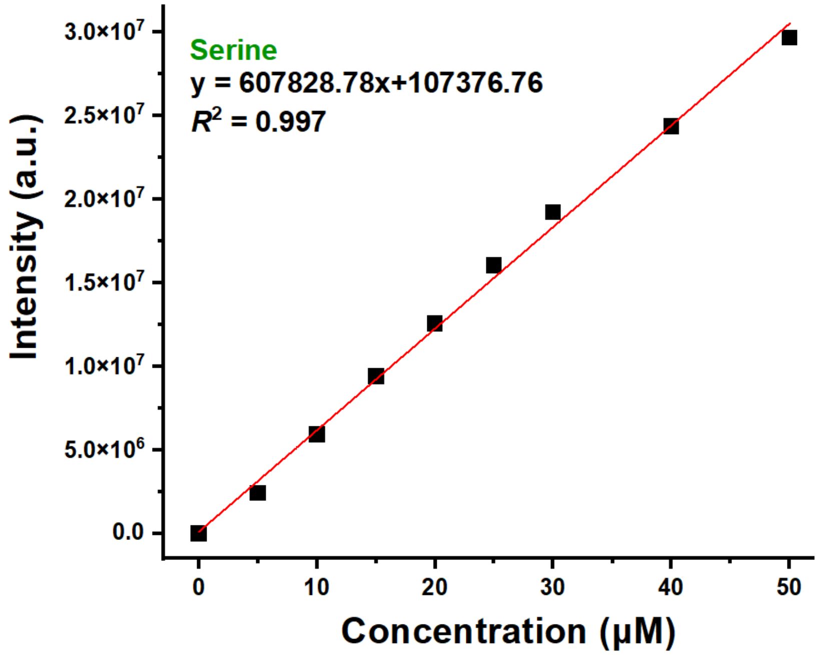

Figure S13. Calibration curve of Ser of the F_nM peptide.

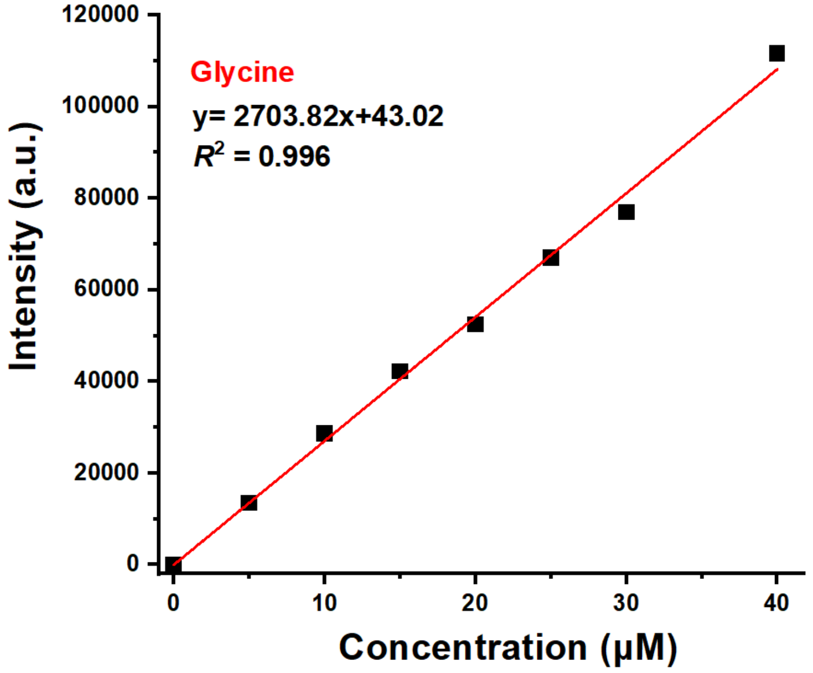

Figure S14. Calibration curve of Gly of the F_nM peptide.

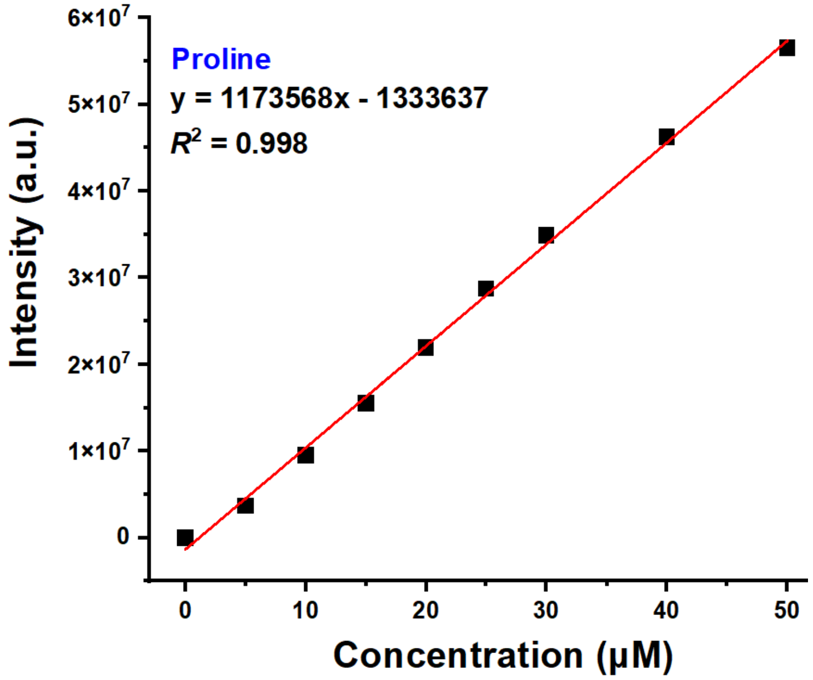

Figure S15. Calibration curve of Pro of the F_nM peptide.

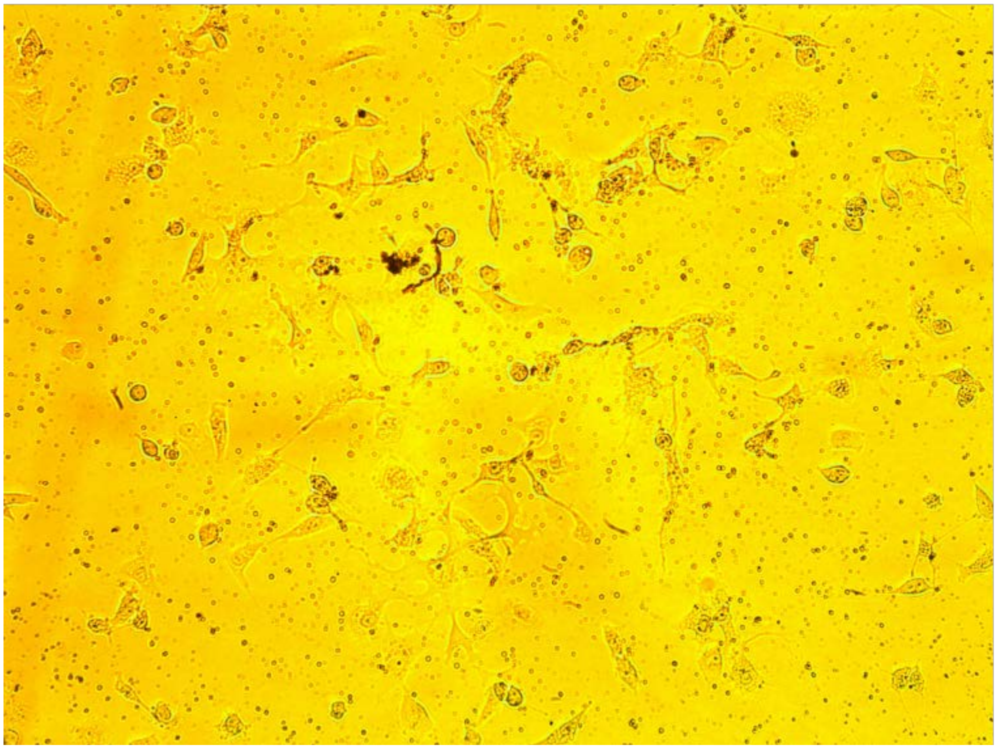

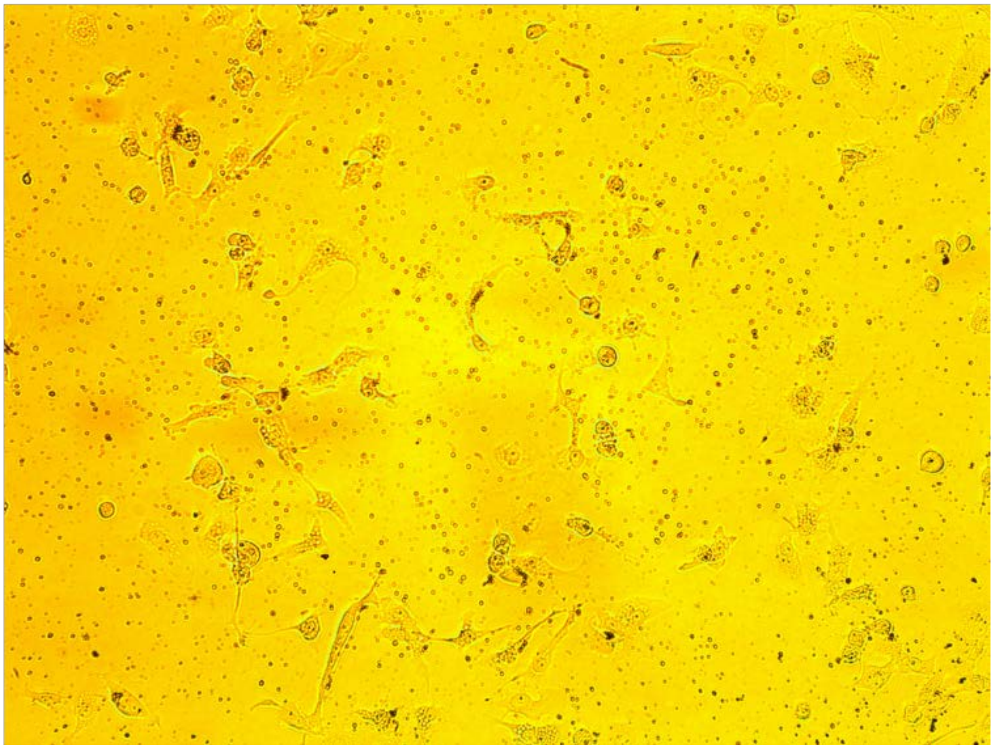

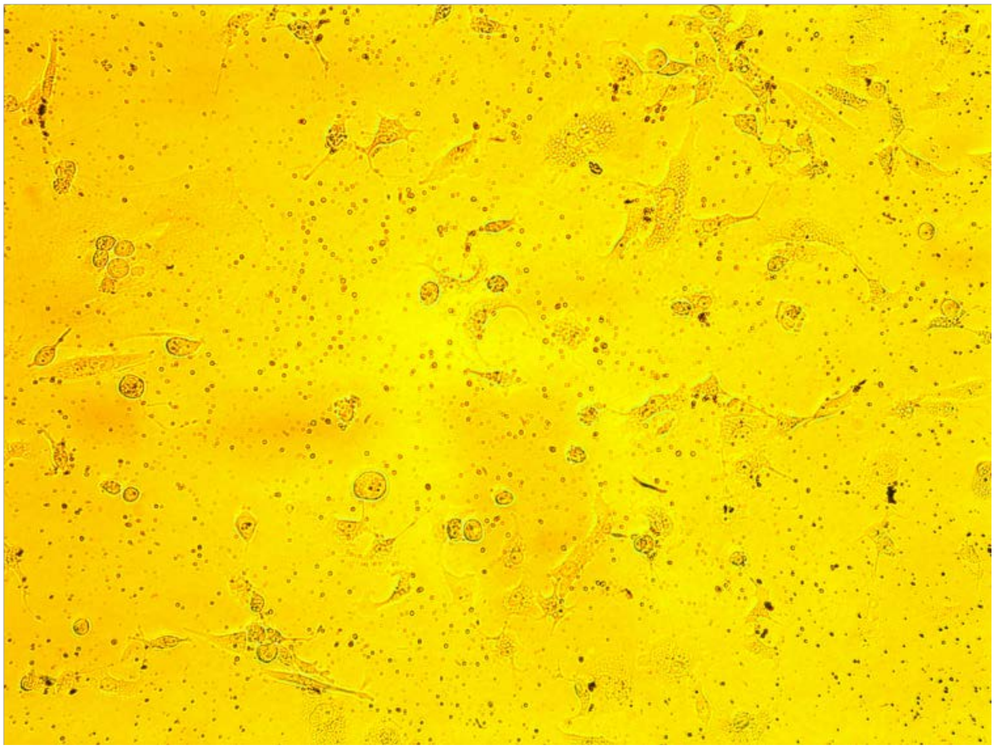

Figure S16. Optical microscopy images of MDA-MB-231 cells incubated for 24 h with F_nM-PEG-S-AuNPs (250 μg Au/mL) supplemented L-15 media at 1:1 ratio, after three washings with PBS.

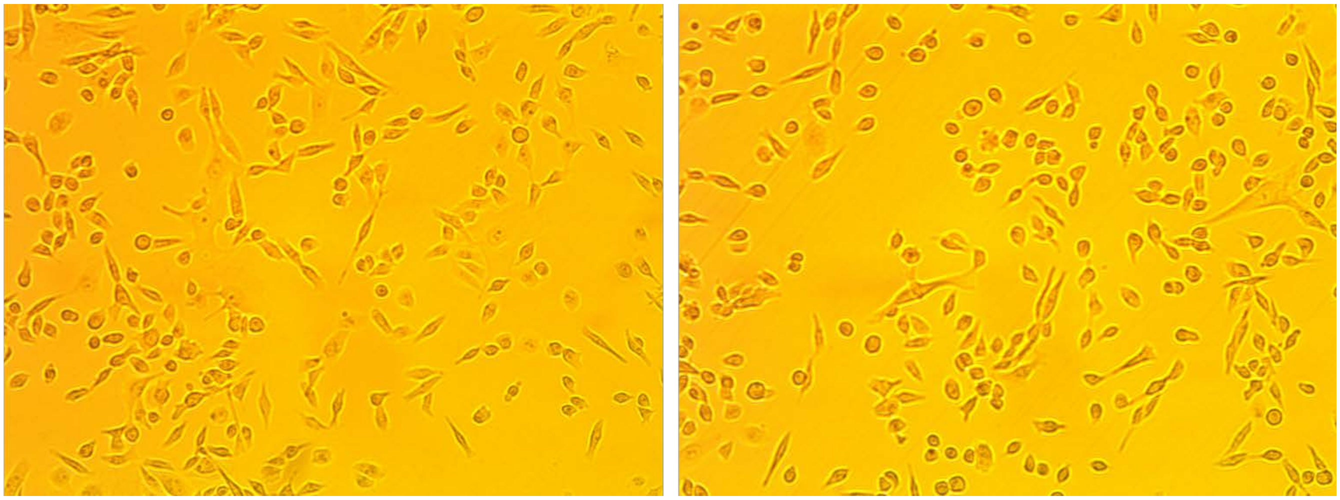

Figure S17. Optical microscopy images of MDA-MB-231 cells incubated for 24 h with L-15 media only at 1:1 ratio, after three washings with PBS.

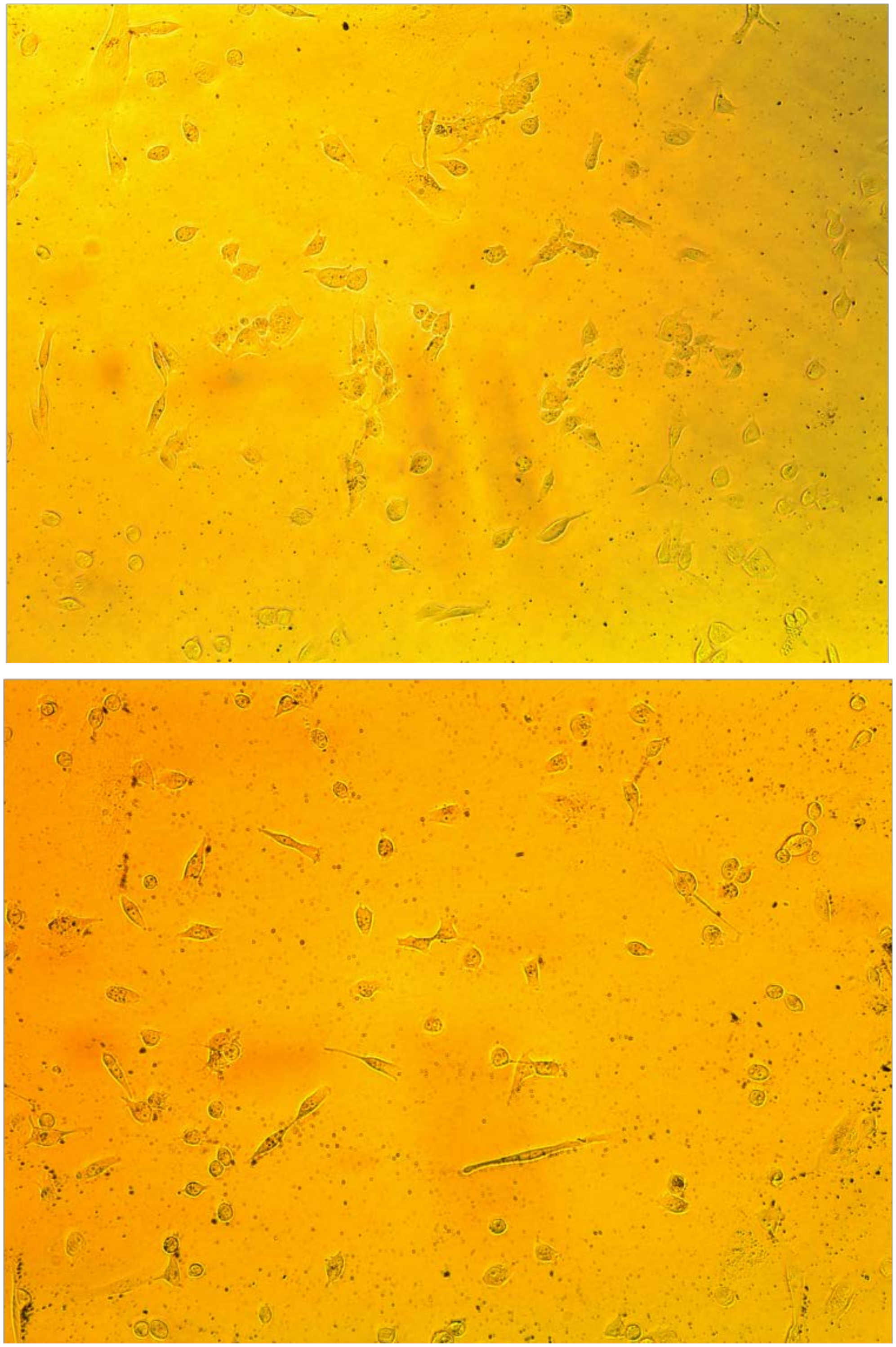

Figure S18. Optical microscopy images of MDA-MB-231 cells incubated for 24 h with mPEG-S-AuNPs (250 μg Au/mL) supplemented L-15 media at 1:1 ratio, after three washings with PBS.

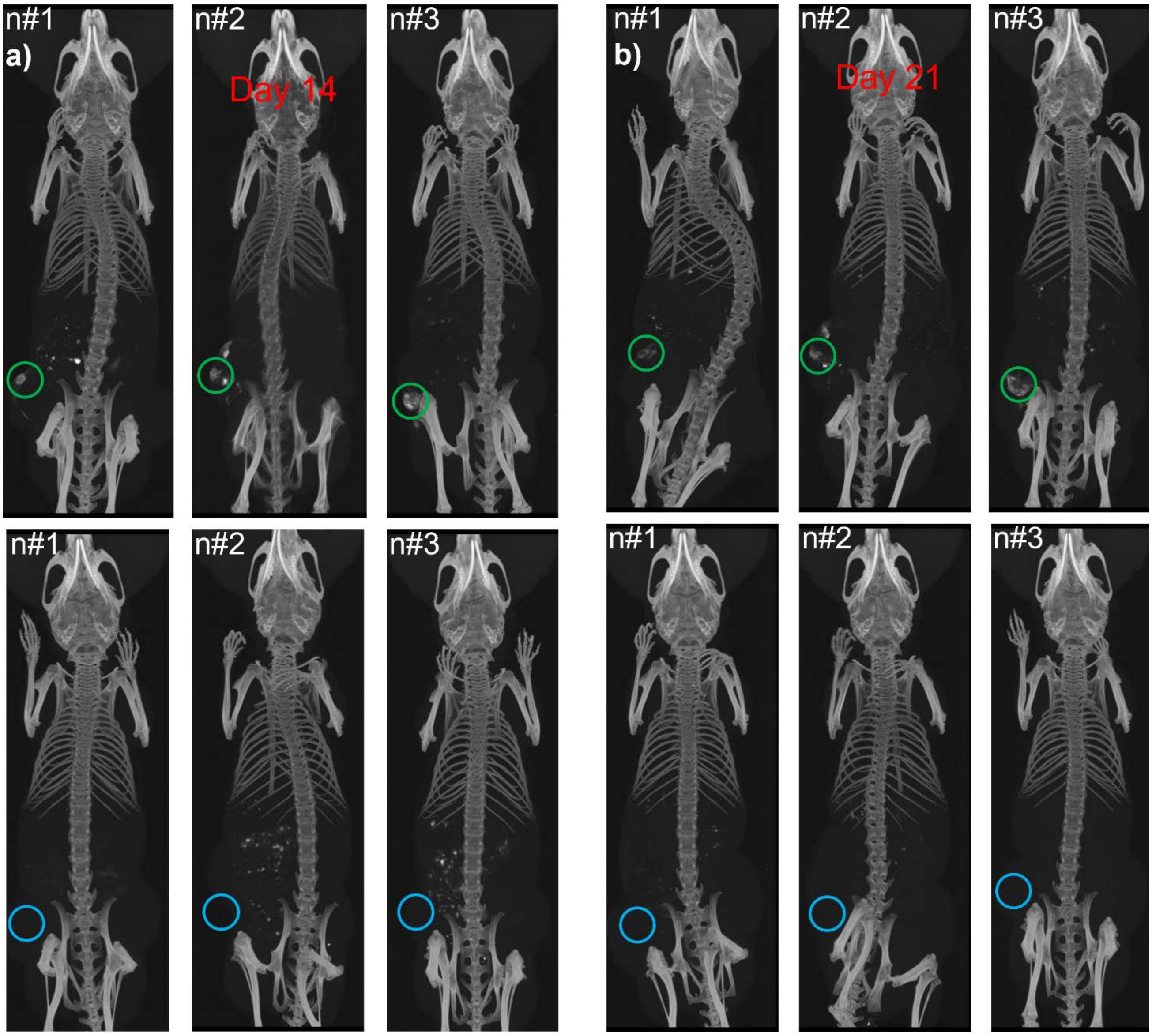

Figure S19. Coronal DE-CT images of mice injected with the F_nM-S-PEG-AuNPs contrast agent (breast tumors shown in green; 0.48 mg Au) and saline (tumors in blue) at (**a**) day 14 and (**b**) day 21. The right tumors were also injected with saline in both cases, but are not shown for image clarity.

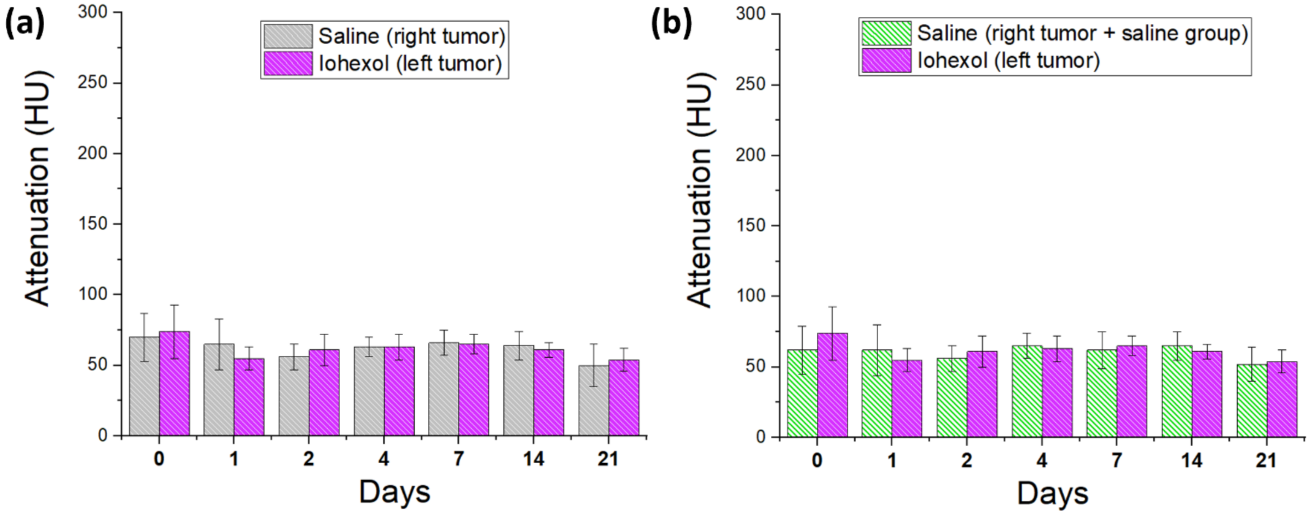

Figure S20. Effect of CT attenuation in breast tumors-bearing mice injected with iohexol contrast agent (left tumor) vs. (**a**) saline (right tumor) within the same animal(s), and (**b**) the merged saline-treated group (left and right tumor of saline group + right tumor of iohexol).

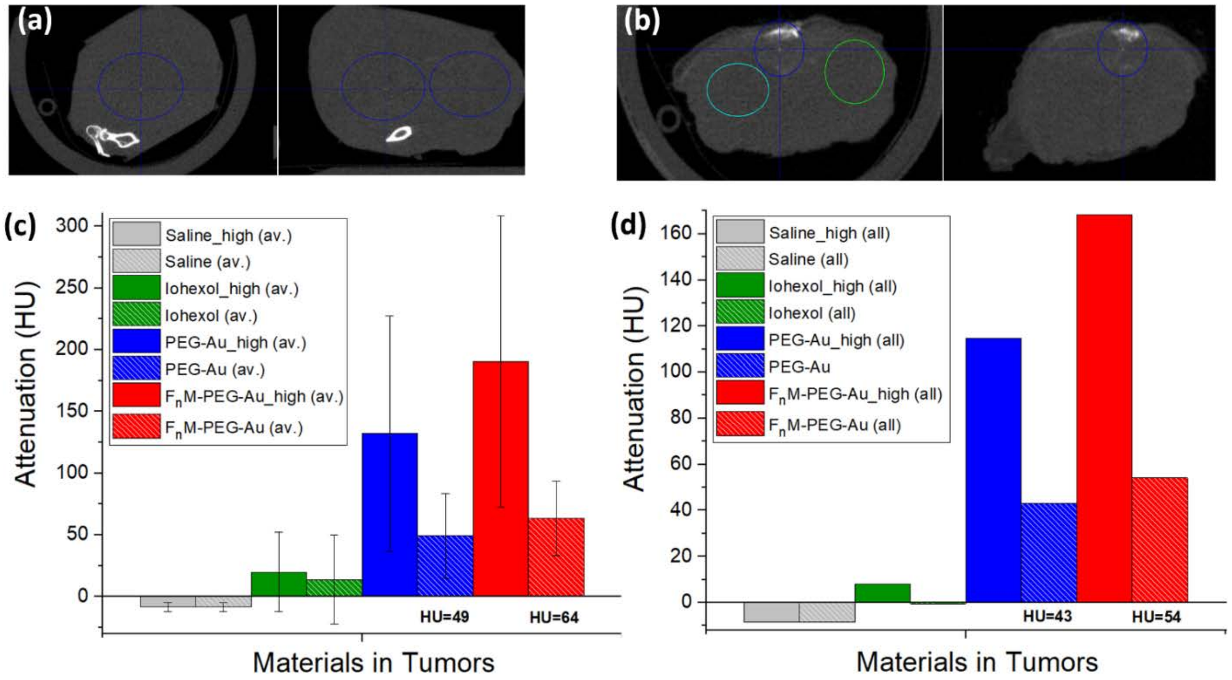

Figure S21. (a) Tumors with encapsulated bone during growth; (b) Three different VOIs within a tumor; (c) Values obtained from these VOIs per animal were *averaged* (white-striped bars). In addition, the VOI with the highest contrast in tumor per animal is reported (fully colored bars); (d) *Median* values of the same VOIs (white-striped bars) as well as the VOI with the highest contrast (fully colored bars). When individual organs with no contrast are scanned and zoomed, non-dense pixels of tissue show negative values. Per manufacturer, water signal has HU ~ 0, while air signal has HU ~ -1000. Animals were administered with saline (grey), iohexol (green), mPEG-AuNPs (blue), and F_nM-S-PEG-AuNPs (red) and sacrificed after day 14.
